# Quantitative phenotype scan statistic (QPSS) reveals rare variant associations with Alzheimer’s disease endophenotypes

**DOI:** 10.1186/s12881-020-01046-6

**Published:** 2020-05-15

**Authors:** Yuriko Katsumata, David W. Fardo

**Affiliations:** 1 Department of Biostatistics, University of Kentucky, Lexington, KY 40536-0082 USA; 2 Sanders-Brown Center on Aging, University of Kentucky, Lexington, KY USA

**Keywords:** QPSS, WGS, ADNI, Localization

## Abstract

**Background:**

Current sequencing technologies have provided for a more comprehensive genome-wide assessment and have increased genotyping accuracy of rare variants. Scan statistic approaches have previously been adapted to genetic sequencing data. Unlike currently-employed association tests, scan-statistic-based approaches can both localize clusters of disease-related variants and, subsequently, examine the phenotype association within the resulting cluster. In this study, we present a novel Quantitative Phenotype Scan Statistic (QPSS) that extends an approach for dichotomous phenotypes to continuous outcomes in order to identify genomic regions where rare quantitative-phenotype-associated variants cluster.

**Results:**

We demonstrate the performance and practicality of QPSS with extensive simulations and an application to a whole-genome sequencing (WGS) study of cerebrospinal fluid (CSF) biomarkers from the Alzheimer’s Disease Neuroimaging Initiative (ADNI). Using QPSS, we identify regions of rare variant enrichment associated with levels of AD-related proteins, CSF Aβ_1–42_ and p-tau_181P_.

**Conclusions:**

QPSS is implemented under the assumption that causal variants within a window have the same direction of effect. Typical self-contained tests employ a null hypothesis of no association between the target variant set and the phenotype. Therefore, an advantage of the proposed competitive test is that it is possible to refine a known region of interest to localize disease-associated clusters. The definition of clusters can be easily adapted based on variant function or annotation.

## Background

Rare variants have become a focus in the recent past. Although genome-wide association studies (GWAS) have been successful in interrogating genetic variants for association with disease, GWAS are performed under the “common disease – common variant” hypothesis positing that common traits are caused by the combination of common variants with a small to moderate effect [1, 2]. GWAS rely on genotyping an array of single nucleotide polymorphisms (SNPs) then imputing ungenotyped variants based on local linkage disequilibrium (LD) derived from reference population haplotypes. Imputation approaches have continually improved and are quite accurate for common variants [3, 4] but are not as reliable for rare variants [5]. Therefore, imputed rare variants are often removed from GWAS analysis. Although GWAS for common variants have revealed numerous susceptibility variants for common diseases, much of the genetic contribution to common diseases remains unexplained [6, 7]. A frequently hypothesized culprit of this missing heritability is the role of rare variants [7, 8].

Next-generation sequencing technologies have allowed for more comprehensive genome-wide approaches, enabling accurate genotyping of rare variants (often defined as a variant with minor allele frequency (MAF) < 1–5%). Consequently, whole-exome sequencing (WES) and whole-genome sequencing (WGS) are ideal approaches to identify novel genes and rare variants associated with complex traits.

Traditional single-variant-based association tests are underpowered to detect rare variant associations unless either or both of the sample size and the effect size are very large [9]. Instead of testing single variants individually, more powerful and computationally efficient approaches for aggregating the effects of rare variants have become the standard for association testing. Many such approaches for testing association between rare variants within a pre-specified region and a disease have been proposed. Burden and variance component tests are two of the most common classes of rare variant analysis methods. Burden tests collapse rare variant effects from a specified region (e.g., gene) using a weighted average of variant counts, whereas variance component tests like the sequence kernel association test (SKAT) use the variance of effect sizes to examine association [10, 11]. Burden tests are more powerful than the SKAT when most of the variants are causal and have the same direction of effect. On the other hand, SKAT is powerful when both risk and protective variants are mixed and when a small proportion of variants are causal [10]. The sheer number of published rare variant methods makes systematic evaluation of relative performance across a spectrum of realistic scenarios challenging [12].

A scan-statistic-based test was introduced into human genetics by Hoh et al. [13] and later adapted to find a window in which rare disease-associated risk variants cluster [1]. The underlying premise is that variants within a functional protein-coding domain may be located in close proximity and may play complementary roles in the genetic mechanisms of a disease. Unlike association tests or other cluster detection analyses, the scan-statistic-based test that we extend in this work can both detect the location of clusters and test for association [14]. Here, the null hypothesis is that rare variants within a certain genetic region/scan window are as likely to confer disease risk as are those outside the window. This approach is generally powerful when there are clusters of disease-related variants with the same direction of association within a selected region/window [14].

In this study, we propose the Quantitative Phenotype Scan Statistic (QPSS), expanding Ionita-Laza et al’s scan-statistic from dichotomized responses to continuous outcomes by way of a normal probability model ([15]; Supplementary Method 1). We then apply QPSS to WGS data from the Alzheimer’s Disease Neuroimaging Initiative (ADNI) with continuous outcomes to identify clusters harboring rare variants associated with Alzheimer’s disease-linked cerebrospinal fluid (CSF) biomarkers.

## Implementation

### QPSS: quantitative phenotype scan statistic

Assume a study comprising *n* subjects, each with a continuous outcome of interest *y*_*i*_ (*i* = 1, …, *n*). Let *m*_*G*_ denote the total number of rare variants in a genetic region of interest *G*, where “rare” is defined by a specified cutoff value (e.g., MAF < 5%). Variants are sorted by physical position in ascending order. We set a sub-window *W* containing *m*_*W*_ variants (*m*_*W*_ < *m*_*G*_) within the genetic region *G* (*i*. *e*., *W* ⊆ *G*). Let *n*_*G*_ be the number of individuals who carry at least one rare variant within the genetic region *G* (*n*_*G*_ ≤ *N*). Of the *n*_*G*_ rare variants carriers, ${n}_{W_{+}}\left(\le {n}_G\right)$ carry a rare variant in the window W. Similarly, ${n}_{W_{-}}\Big(={n}_G-{n}_{W_{+}}$) do not carry a rare variant within window W. We can then partition the maximum likelihood estimate of the trait variance ${\sigma}_W^2$ among the *n*_*G*_ rare variant carriers as

$$ {\hat{\sigma}}_W^2=\frac{1}{n_G}\left\{\sum \limits_{i\in \left\{1,\dots, {n}_{W_{+}}\right\}}{\left({y}_i-{\hat{\mu}}_{W_{+}}\right)}^2+\sum \limits_{i\in \left\{1,\dots, {n}_{W_{-}}\right\}}{\left({y}_i-{\hat{\mu}}_{W_{-}}\right)}^2\right\} $$

where

$$ {\hat{\mu}}_{W_{+}}=\frac{1}{n_{W_{+}}}\sum \limits_{i\in \left\{1,\dots, {n}_{W_{+}}\right\}}{y}_i $$

$$ {\hat{\mu}}_{W_{-}}=\frac{1}{n_{W_{-}}}\sum \limits_{i\in \left\{1,\dots, {n}_{W_{-}}\right\}}{y}_i $$

Under the null hypothesis that variants within the sub-window *W* are equally as likely to correlate with the disease outcome that those outside the window (but still within the region of interest *G*), the outcome variance should be similar between the ${n}_{W_{-}}$ and ${n}_{W_{+}}$ subjects so that the pooled variance would equal the overall variance, that is,

$$ {\sigma}_W^2={\sigma}_0^2 $$

where ${\sigma}_0^2$ is the variance under the null hypothesis and its maximum likelihood estimate is

$$ {\hat{\sigma}}_0^2=\frac{1}{n_G}\sum \limits_{i\in \left\{1,\dots, {n}_G\right\}}{\left({y}_i-{\hat{\mu}}_0\right)}^2 $$

$$ {\hat{\mu}}_0=\frac{1}{n_G}\sum \limits_{i\in \left\{1,\dots, {n}_G\right\}}{y}_i $$

Under the alternative hypothesis, we expect the outcome values of the ${n}_{W_{+}}$ subjects carrying a rare variant within *W* to be more similar to each other than to the rare variant carrying subjects whose variants are all outside *W*, that is ${\sigma}_W^2<{\sigma}_0^2$. Using these definitions, we calculate the following log likelihood ratio test statistic

$$ \ln {\hat{LR}}_W=\frac{n_G}{2}\ln \frac{{\hat{\sigma}}_0^2}{{\hat{\sigma}}_W^2} $$

for the window *W* (see Supplementary Method 1 for full details).

In order to distinguish between risk and protective clusters, we can adjust the log likelihood ratio test statistic based on the estimated effect direction. For instance, Kulldorff et al. suggested the simple indicator function $I\left({\hat{\mu}}_{W_{+}}>{\hat{\mu}}_{W_{-}}\right)$ for risk clusters with high values of the outcome, which effectively removes from consideration any window *W* where the trait mean among subjects in *W*_+_ is less that that for subjects in *W*_−_. Similarly, $I\left({\hat{\mu}}_{W_{+}}<{\hat{\mu}}_{W_{-}}\right)$ can be used for protective clusters with low values of the outcome [15]. To evaluate both positive and negative associations of clusters with the phenotype simultaneously, we can employ the sign function $\mathit{\operatorname{sgn}}\left({\hat{\mu}}_{W_{+}}-{\hat{\mu}}_{W_{-}}\right)$, that is, an indicator of +1 for ${\hat{\mu}}_{W_{+}}>{\hat{\mu}}_{W_{-}}$ and −1 for ${\hat{\mu}}_{W_{+}}<{\hat{\mu}}_{W_{-}}$. The window harboring rare variants associated with a phenotype can be identified as the window that maximizes the log likelihood ratio, i.e.,

$$ \underset{W}{\max}\left|\ln {\hat{LR}}_W\times \mathit{\operatorname{sgn}}\left({\hat{\mu}}_{W_{+}}-{\hat{\mu}}_{W_{-}}\right)\right|. $$

The null distribution of the test statistic is unknown; thus, *p*-values are calculated by a permutation approach [16]. To minimize computation time, we applied a GPD approximation [17] (see Supplementary Method 2 for full details) for estimating p-value of permutation test in the application study.

### Simulation study

#### Genotype data

We generated 10,000 haplotypes of a 1 Mb genomic region from a European population as implemented in the software Cosi2 (https://software.broadinstitute.org/mpg/cosi2/) [18]. We randomly extracted haplotype pairs to create genotypes for sample sizes (n) of 500 and 1000. We then removed common variants (MAF > 0.05) and singletons.

#### Phenotype data

We considered three cluster sizes: small (200 basepairs (bp)) and moderate (500 bp), both of which have consecutively located disease-associated rare variants; and large (2 kb) where 20% of rare variants are disease-related (thus, not consecutive). In each of the clusters, we randomly chose a start position and then generated quantitative phenotypes from the model

$$ {y}_i=\sum \limits_{j=1}^{m_C}{\beta}_j{g}_{ij}+{\varepsilon}_i $$

where *β*_*j*_ = *c*|log_10_*MAF*_*j*_|, *m*_*C*_ is the number of variants in the cluster, *g*_*ij*_ is the additively-coded genotype (*g*_*ij*_ = 0, 1, 2 according to the minor allele count) for the *i* th individual at the *j* th variant (*j* = 1, …, *m*_*C*_), and *ε*_*i*_ is the error for the *i* th individual generated from a standard normal (i.e., *ε*_*i*_ ∼ *N*(0, 1)). We set *c* = 0.2, *c* = 0.4 and *c* = 0.6 for the empirical power simulations, and *c* = 0 for the type I error simulations. For each scenario, we generated 1000 simulation replicates.

**Fig. 1:**
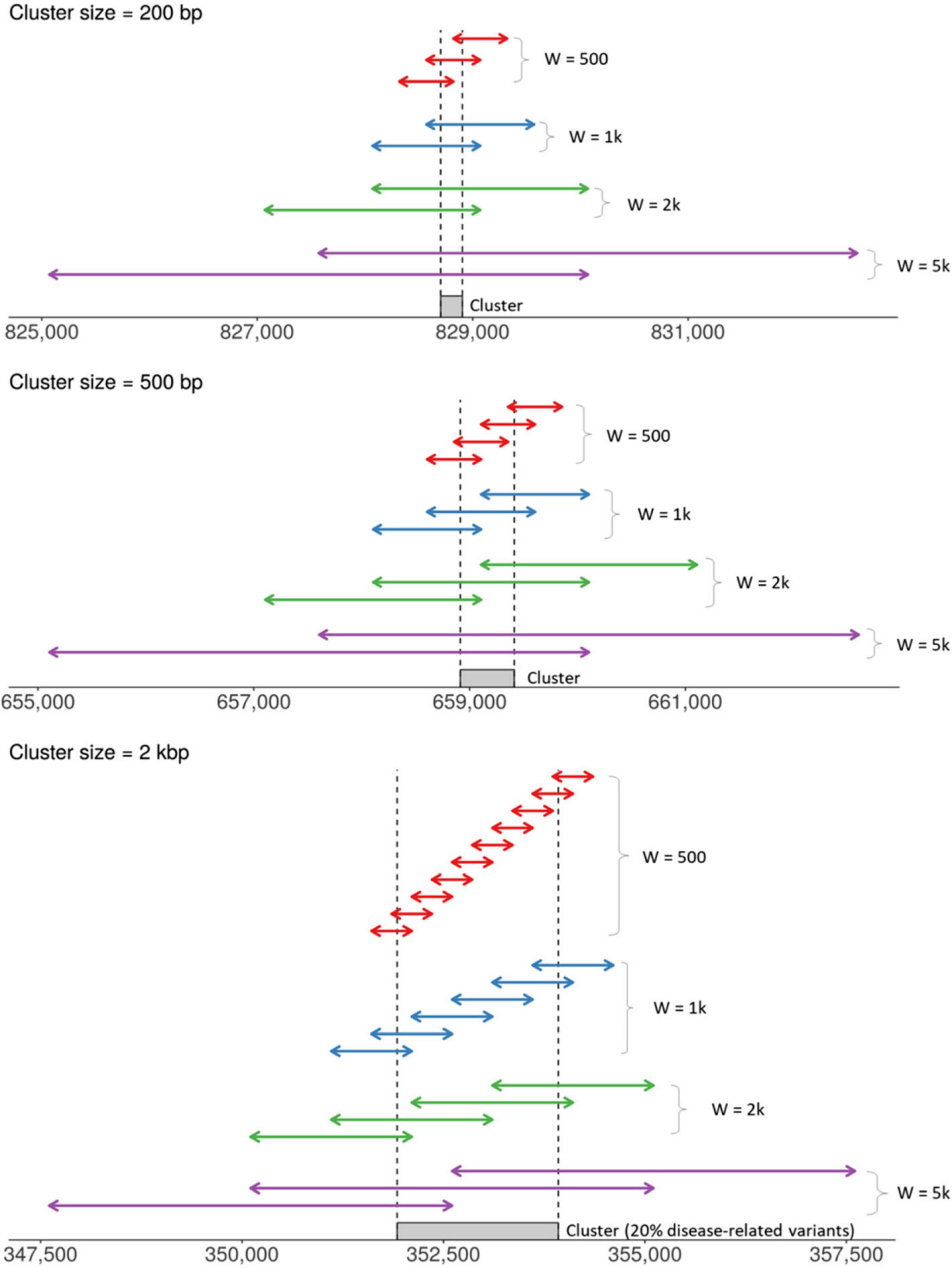
Cluster and window positions for type I error and power evaluations in each scenario

Specification of the overall genetic region *G* and the subwindows *W* are flexible. To find a window that maximizes the log likelihood ratio, we thus employed a sliding window approach considering several different window sizes. We used windows sizes of 5 k, 2 k, 1 k, and 500 bp, and then slid each of those windows by half its respective size (i.e., by 2.5 k, 1 k, 500, and 250 bp). These scenarios are depicted both graphically and via a table (Fig. 1 and Supplementary Table 1). To provide a better sense of the effect sizes examined, the means of the simulated phenotypes (Supplementary Figure 1) and the estimates of genetic heritability (Supplementary Figure 2) are shown for each scenario.

## Results

### Type I error simulation results

The empirical type I error rates were calculated as the proportion of *p*-values less than or equal to the corresponding Bonferroni-adjusted significance level (*α*^∗^ = *α*/ the number of examined sliding windows in the region *G*) for the window in which $\ln {\hat{LR}}_W$ was maximized. As shown in Table 1, the type I error rates were acceptable in all scenarios.

**Table 1:** Type I error estimates with Bonferroni correction

	Window size/sliding window size (m = the number of sliding windows in the region *G*)			
	5 k/2.5 k (m = 400)	2 k/1 k (m = 1000)	1 k/500 (m = 2000)	500/250 (m = 4000)
*n* = 500				
α = 0.05	0.047	0.054	0.053	0.051
α = 0.01	0.008	0.016	0.007	0.011
*n* = 1000				
α = 0.05	0.051	0.052	0.051	0.033
α = 0.01	0.013	0.009	0.012	0.008

Empirical type I error rates were estimated as proportion of p-values less than or equal to the corresponding Bonferroni-corrected significance level (α* = α /m) for the window where $\ln {\hat{LR}}_W$ is maximized

#### Power simulation results

Power was calculated as the proportion of simulation replicates with an empirical *p*-value (corresponding to the window with maximum value of $\ln {\hat{LR}}_W$) reaching Bonferroni-adjusted significance. Supplementary Figures 3 to 20 show the means of $\ln {\hat{LR}}_W$ (over the 1000 simulation replicates) in each of the windows. Supplementary Tables 2 and 3 show the number of the targeted sliding windows (those containing true disease-related variants) with achieved the maximum value of $\ln {\hat{LR}}_W$. Figure 2 shows the empirical powers for the targeted sliding windows under the alternative hypothesis in each scenario when all disease-related variants had positive associations with the phenotype.

**Fig. 2:**
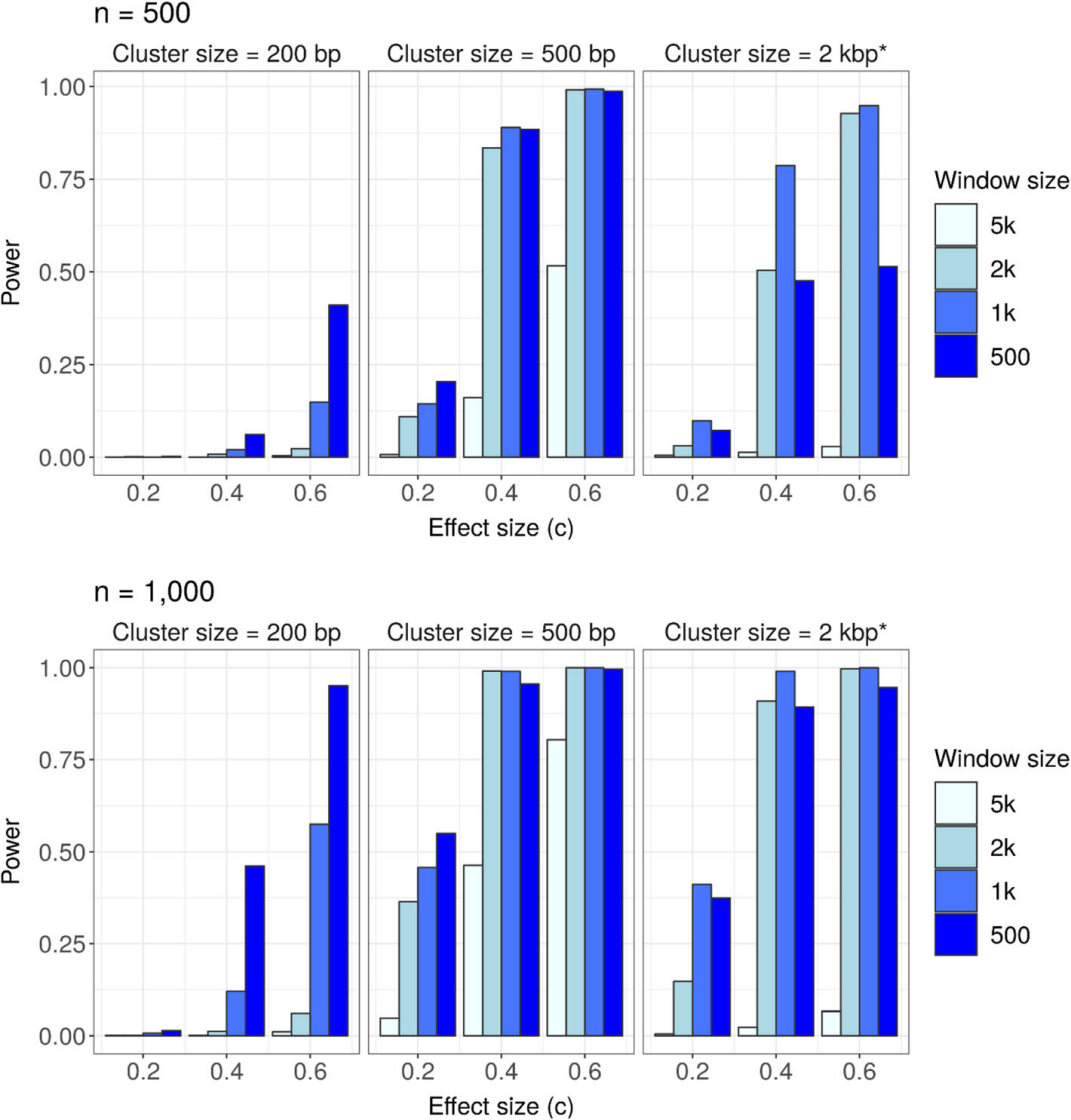
Empirical power for QPSS to detect any targeted sliding window (one containing true disease-related variants). ^*^ Cluster size = 2 kbp contains 20% disease-related variants

When the effect size and the cluster size were small (c = 0.2 and cluster size = 200 bp), the proposed QPSS had low power in all scanning windows. In other scenarios, power depends on the scanning window size relative to the cluster size. When the scanning window is too large compared to the cluster size, power can decrease considerably. On the other hand, when the scanning window size is smaller than the size of the variant-containing cluster, multiple sliding windows can overlap the outcome-related cluster; however, it was hard to identify the window most likely to harbor risk variants. Not surprisingly, the best scenario was when the scanning window is of similar length to the true cluster.

### Application to Alzheimer’s Disease Neuroimaging Initiative (ADNI)

Data used in the preparation of this article were obtained from the ADNI database (adni.loni.usc.edu). The ADNI was launched in 2003 as a public-private partnership, led by Principal Investigator Michael W. Weiner, MD. The primary goal of ADNI has been to test whether serial magnetic resonance imaging (MRI), positron emission tomography (PET), other biological markers, and clinical and neuropsychological assessment can be combined to measure the progression of mild cognitive impairment (MCI) and early AD. We retrieved baseline data on CSF amyloid β 1–42 (Aβ_1–42_) and phosphorylated tau at the threonine 181 (p-tau_181P_) levels measured at the ADNI Biomarker Core laboratory at the University of Pennsylvania Medical Center [19, 20] and single nucleotide variants (SNVs) from WGS data sequenced on the Illumina HiSeq2000 using paired-end 100-bp reads. We used data from 538 subjects aged 65 years or older at the time when the baseline CSF sample was drawn.

The apolipoprotein E (*APOE*) gene, located on chromosome 19q13.32, is involved in Aβ_1–42_ deposition [21], and the microtubule-associated protein tau (*MAPT*) gene, located on chromosome 17q21.31, encodes tau protein. Thus, we first examined the associations of genes on chromosome 19 with CSF Aβ_1–42_ levels and of genes on chromosome 17 with CSF p-tau_181P_ levels using common gene-based association tests including the burden test, SKAT, and SKAT-O. The start and end gene positions on hg19 assembly of each gene were obtained in the UCSC Genome Browser (https://genome.ucsc.edu/) [22]. Several genes close to *APOE* (19: 45,409,039 – 45,412,650) had significant associations (Bonferroni corrected for 1639 genes) with CSF Aβ_1–42_ levels: *PVRL2* (19: 45,349,393 – 45,392,485; SKAT *p* = 1.87 × 10^− 5^); *TOMM40* (19: 45,394,477 – 45,406,946; SKAT *p* = 2.81 × 10^− 9^ and SKAT-O *p* = 1.97 × 10^− 8^); *APOC1* (19:45,417,921 – 45,422,606; SKAT *p* = 1.16 × 10^− 8^ and SKAT-O *p* = 3.47 × 10^− 8^) (Supplementary Figure 21). No genes that reached Bonferroni-adjusted significance in the gene-based association with CSF p-tau_181P_ (Supplementary Figure 22).

Next, we evaluated whether QPSS detected clusters on chromosomes 19 and 17 associated with CSF Aβ_1–42_ and p-tau_181P_ levels, respectively. We set the *APOE* and *MAPT* gene lengths ±10 Mbp as the large genetic region *G*, window sizes 5 k/2 k/1 k/500 bp and sliding length 2.5 k/1 k/500/250 bp. All analyses were restricted to rare variants (MAF < 0.05). The total numbers of windows evaluated were m = 133,687 in the *APOE* region and m = 126,623 in the *MAPT* region; therefore the Bonferroni-corrected significance levels were α = 0.05/133,687 ≈ 3.74 × 10^−7^ and α = 0.05/126,623 ≈ 3.95 × 10^−7^, respectively.

**Fig. 3:**
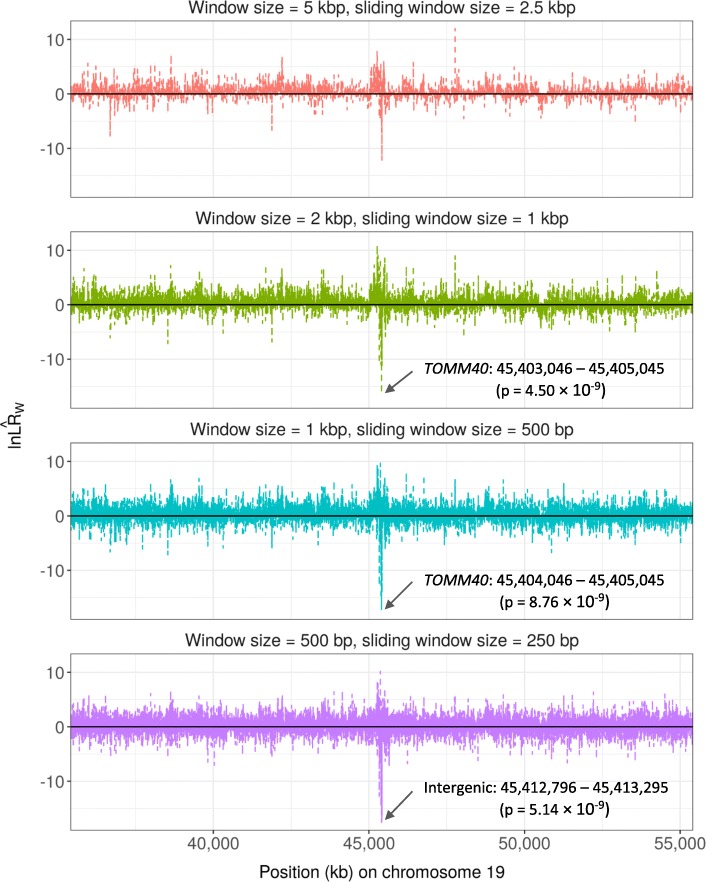
Plots of $\ln {\hat{LR}}_W$ for the associations between rare variants around *APOE* (± 10 M bp) located on chromosome 19 and log-transformed CSF amyloid β 1–42 levels in ADNI. Positive $\ln {\hat{LR}}_W$ represents test statistics for ${\hat{\mu}}_{W_{+}}>{\hat{\mu}}_{W_{-}}$ and negatives for ${\hat{\mu}}_{W_{+}}<{\hat{\mu}}_{W_{-}}$

**Fig. 4:**
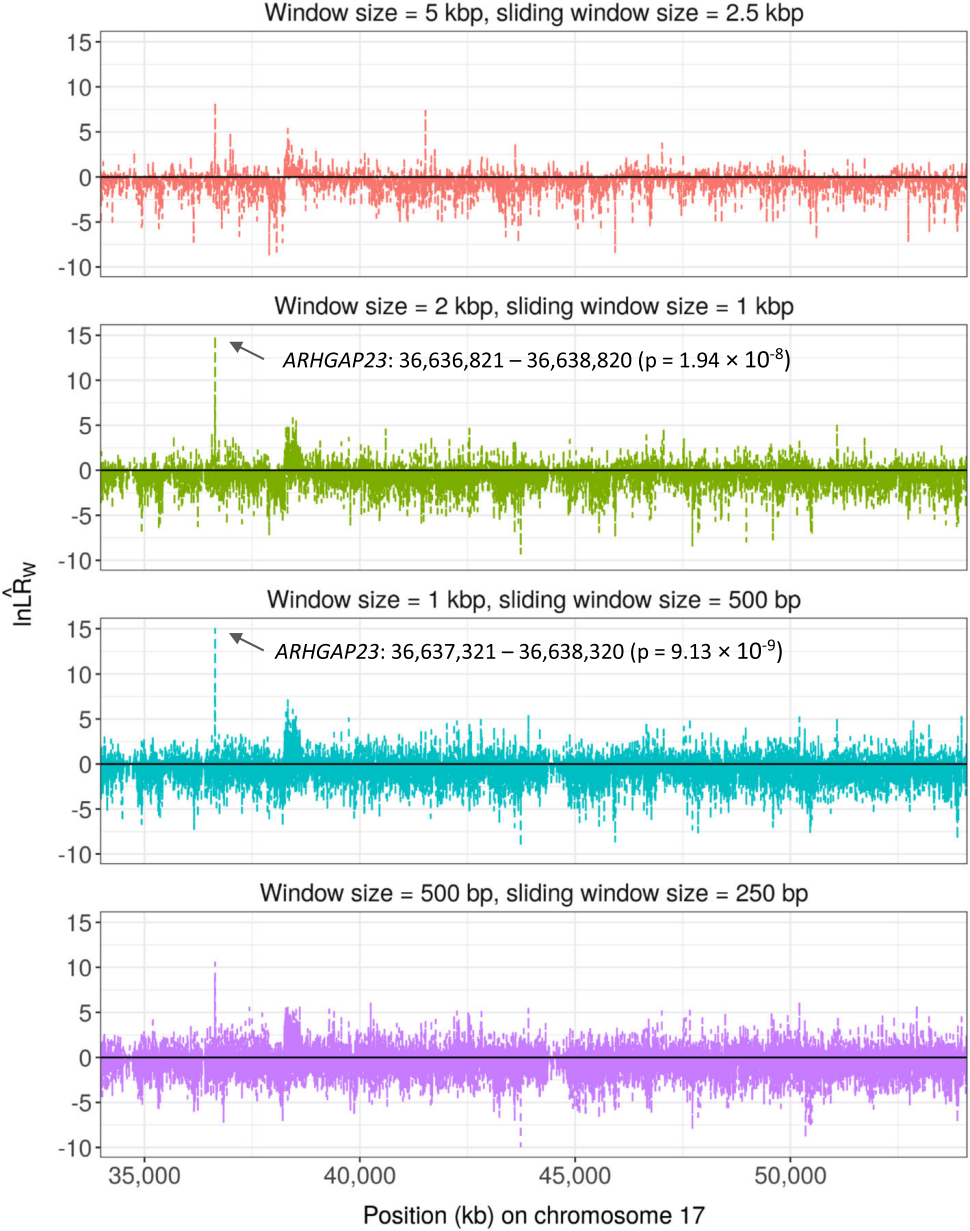
Plots of $\ln {\hat{LR}}_W$ for the associations between rare variants around *MAPT* (± 10 M bp) located on chromosome 17 and log-transformed CSF phosphorylated tau levels in ADNI. Positive $\ln {\hat{LR}}_W$ represents test statistics for ${\hat{\mu}}_{W_{+}}>{\hat{\mu}}_{W_{-}}$ and negatives for ${\hat{\mu}}_{W_{+}}<{\hat{\mu}}_{W_{-}}$

Figure 3 and Supplementary Figure 23 show the $\ln {\hat{LR}}_W$ values and *p*-values for windows in the associations of rare variants in the *APOE* region ±10 Mbp with CSF Aβ_1–42_ levels. The positive $\ln {\hat{LR}}_W$ values represent ${\hat{\mu}}_{W_{+}}>{\hat{\mu}}_{W_{-}}$ for clusters associated with high values of the outcome and the negative one for ${\hat{\mu}}_{W_{+}}<{\hat{\mu}}_{W_{-}}$ for clusters associated with low values of the outcome. Windows that maximized $\ln {\hat{LR}}_W$ and resulted in a significant permutation-based p-values were 45,403,046 – 45,405,045 in *TOMM40* for the scanning window size of 2 kbp (*p* = 4.50 × 10^− 9^), 45,404,046 – 45,405,045 in *TOMM40* for the scanning window size of 1 kbp (*p* = 8.76 × 10^− 9^), and 45,412,796 – 45,413,295 in the intergenic region 150 bp away from *APOE* 3′ UTR (*p* = 5.14 × 10^− 9^). For the associations with CSF p-tau_181P_, significant windows were 36,636,821 – 36,638,820 for the scanning window size of 2 kbp (*p* = 1.94 × 10^− 8^) and 36,637,321 – 36,638,320 the scanning window size of 1 kbp (*p* = 9.13 × 10^− 9^), both of which were in *ARHGAP23* (Fig. 4 and Supplementary Figure 24).

## Discussion

We showed the performance and practicality of QPSS with extensive simulations and in application to a WGS dataset with CSF biomarkers from ADNI. We identified regions enriched with rare variants in *TOMM40* and the surrounding intergenic region that were associated with decreased CSF Aβ_1–42_ levels and a cluster in *ARHGAP23* with rare variants associated with increased CSF p-tau_181P_ levels. The window 45,403,046 – 45,405,045 in *TOMM40* and the window 45,412,796 – 45,413,295 in the intergenic region contained 13 rare variants and two rare variants, respectively. These windows successfully captured three top loci associated with decreased CSF Aβ_1–42_ levels (Supplementary Tables 4 and 5 and Supplementary Figure 25). Unlike *TOMM40*, *ARHGAP23* went undetected using commonly-used gene-based tests (i.e., burden test, SKAT, and SKAT-O). Using a 1 k bp window (and corresponding 500 bp slide), the window 36,637,321 – 36,638,320 in *ARHGAP23* was the most likely to harbor a cluster of variants associated increased CSF p-tau_181P_ levels, which was significant after Bonferroni correction (*p* = 9.13 × 10^− 9^ using generalized Pareto distribution (GPD) approximation). There were three rare variants in the implicated window in *ARHGAP23*; each minor allele associated with increased CSF p-tau_181P_ levels (Supplementary Table 6). The association between *ARHGAP23* and CSF p-tau_181P_ levels is novel and warrants attempts at replication in future work. Notably, *ARHGAP23* is located ~ 7 Mb from the *MAPT* gene suggesting that this is an independent signal.

## Conclusions

QPSS is implemented under the assumption, similar to burden tests, that causal variants within a window have the same direction of effect. However, there is a difference in the nature of the tested hypotheses between these methods. The null hypothesis of the competitive tests, like our proposed method, is that associations between the phenotype and the set of variants within a specified window are the same as those outside the window. Typical self-contained tests employ a null hypothesis of no association between the target variant set and the phenotype. Therefore, an advantage of the proposed competitive test is that it is possible to refine a known region of interest to localize disease-associated clusters. Note that the definition of clusters can be easily adapted based on variant function or annotation. A limitation of these approaches is the possibility of population structure confounding as the proposed method does not take into account covariate adjustment. We aim to address this limitation in future work.

## Availability and requirements

**Project name:** Quantitative Phenotype Scan Statistic (QPSS).

**Project home page:** https://github.com/kyka222/QPSS

**Operating system(s):** Platform independent.

**Programming language:** Python2.

**Other requirements:** PLINK 1.9, R 2.10 or higher, R package goft.

**License**: Free academic research use.

**Any restrictions to use by non-academics:** License required.

## Supplementary information

**Additional file 1: Supplementary Method 1**. Scan statistics for the normal probability model developed by Kulldorff et al. [1]. **Supplementary Method 2**. Computing empirical *p*-values based on permutation test and approximation by a generalized Pareto distribution described by Knijnenburg et al. [2]. **Table S1**. Simulation scenario for type I error and power evaluations. **Table S2**. Frequency of the number of targeted sliding windows that produced the maximum value of $\ln {\hat{LR}}_W$ (*n* = 500). **Table S3**. Frequency of the number of targeted sliding windows that produced the maximum value of $\ln {\hat{LR}}_W$ (*n* = 1000). **Table S4**. Single variant associations on the significant window of TOMM40 (45,403,046 – 45,405,045) with log-transformed cerebrospinal fluid amyloid β 1–42 levels in ADNI. **Table S5**. Single variant associations on the significant window of intergenic region (45,412,796 – 45,413,295) with log-transformed cerebrospinal fluid amyloid β 1–42 levels in ADNI. **Table S6**. Single variant associations on the significant window of ARHGAP23 (36,637,321 – 36,638,320) with log-transformed cerebrospinal fluid phosphorylated tau levels in ADNI. **Figure S1**. Mean of continuous phenotype y in each scenario. **Figure S2**. Estimate of heritability in each scenario. **Figure S3**. Mean of $\ln {\hat{LR}}_W$ for *n* = 500, cluster size = 200 bp, and effect size c = 0.2. Each point represents the center position of each of the windows, and the blue vertical line indicates the center of the cluster position. **Figure S4**. Mean of $\ln {\hat{LR}}_W$ for *n* = 500, cluster size = 200 bp, and effect size c = 0.4. Each point represents the center position of each of the windows, and the blue vertical line indicates the center of the cluster position. **Figure S5**. Mean of $\ln {\hat{LR}}_W$ for *n* = 500, cluster size = 200 bp, and effect size c = 0.6. Each point represents the center position of each of the windows, and the blue vertical line indicates the center of the cluster position. **Figure S6**. Mean of $\ln {\hat{LR}}_W$ for *n* = 500, cluster size = 500 bp, and effect size c = 0.2. Each point represents the center position of each of the windows, and the blue vertical line indicates the center of the cluster position. **Figure S7**. Mean of $\ln {\hat{LR}}_W$ for *n* = 500, cluster size = 500 bp, and effect size c = 0.4. Each point represents the center position of each of the windows, and the blue vertical line indicates the center of the cluster position. **Figure S8**. Mean of $\ln {\hat{LR}}_W$ for *n* = 500, cluster size = 500 bp, and effect size c = 0.6. Each point represents the center position of each of the windows, and the blue vertical line indicates the center of the cluster position. **Figure S9**. Mean of $\ln {\hat{LR}}_W$ for n = 500, cluster size = 2 kbp (containing 20% disease-related variants), and effect size c = 0.2. Each point represents the center position of each of the windows, and the blue vertical line indicates the center of the cluster position. **Figure S10**. Mean of $\ln {\hat{LR}}_W$ for n = 500, cluster size = 2 kbp (containing 20% disease-related variants), and effect size c = 0.4. Each point represents the center position of each of the windows, and the blue vertical line indicates the center of the cluster position. **Figure S11**. Mean of $\ln {\hat{LR}}_W$ for n = 500, cluster size = 2 kbp (containing 20% disease-related variants), and effect size c = 0.6. Each point represents the center position of each of the windows, and the blue vertical line indicates the center of the cluster position. **Figure S12**. Mean of $\ln {\hat{LR}}_W$ for *n* = 1000, cluster size = 200 bp, and effect size c = 0.2. Each point represents the center position of each of the windows, and the blue vertical line indicates the center of the cluster position. **Figure S13**. Mean of $\ln {\hat{LR}}_W$ for *n* = 1000, cluster size = 200 bp, and effect size c = 0.4. Each point represents the center position of each of the windows, and the blue vertical line indicates the center of the cluster position. **Figure S14**. Mean of $\ln {\hat{LR}}_W$ for *n* = 1000, cluster size = 200 bp, and effect size c = 0.6. Each point represents the center position of each of the windows, and the blue vertical line indicates the center of the cluster position. **Figure S15**. Mean of $\ln {\hat{LR}}_W$ for *n* = 1000, cluster size = 500 bp, and effect size c = 0.2. Each point represents the center position of each of the windows, and the blue vertical line indicates the center of the cluster position. **Figure S16**. Mean of $\ln {\hat{LR}}_W$ for n = 1000, cluster size = 500 bp, and effect size c = 0.4. Each point represents the center position of each of the windows, and the blue vertical line indicates the center of the cluster position. **Figure S17**. Mean $\ln {\hat{LR}}_W$ for *n* = 1000, cluster size = 500 bp, and effect size c = 0.6. Each point represents the center position of each of the windows, and the blue vertical line indicates the center of the cluster position. **Figure S18**. Mean of $\ln {\hat{LR}}_W$ for n = 1000, cluster size = 2 kbp (containing 20% disease-related variants), and effect size c = 0.2. Each point represents the center position of each of the windows, and the blue vertical line indicates the center of the cluster position. **Figure S19**. Mean of $\ln {\hat{LR}}_W$ for n = 1000, cluster size = 2 kbp (containing 20% disease-related variants), and effect size c = 0.4. Each point represents the center position of each of the windows, and the blue vertical line indicates the center of the cluster position. **Figure S20**. Mean of $\ln {\hat{LR}}_W$ for n = 1000, cluster size = 2 kbp (containing 20% disease-related variants), and effect size c = 0.6. Each point represents the center position of each of the windows, and the blue vertical line indicates the center of the cluster position. **Figure S21**. Gene-based associations between rare variants located on chromosome 19 and log-transformed CSF amyloid β 1–42 levels in ADNI using the burden test, SKAT, and SKAT-O. The red horizontal line indicates the significance level with Bonferroni correction (α = 0.05/the number of genes on chromosome 19). **Figure S22**. Gene-based associations between rare variants located on chromosome 17 and log-transformed CSF phosphorylated tau levels in ADNI using the burden test, SKAT, and SKAT-O. The red horizontal line indicates the significance level with Bonferroni correction (α = 0.05/the number of genes on chromosome 17). **Figure S23**. QPSS p-values computed by the permutation with generalized Pareto distribution approximation for the associations between rare variants around APOE (± 10 Mbp) located on chromosome 19 and log-transformed CSF amyloid β 1-42 in ADNI. **Figure S24**. QPSS p-values computed by the permutation with generalized Pareto distribution approximation for the associations between rare variants around MAPT (± 10 Mbp) located on chromosome 17 and log-transformed CSF phosphorylated tau levels in ADNI. **Figure S25**. Single variant associations between rare variants around APOE (± 10 Mbp) located on chromosome 19 and log-transformed CSF amyloid β 1- 42 in ADNI.

## Data Availability

Cerebrospinal fluid (CSF) biomarkers and whole genome sequences data from the Alzheimer’s Disease Neuroimaging Initiative (ADNI) were downloaded from the Image & Data Archive at the Laboratory of Neuro Imaging (https://ida.loni.usc.edu/login.jsp). We have received administrative approval for access to the ADNI database.

## Abbreviations

ADNI: Alzheimer’s Disease Neuroimaging Initiative
CSF: Cerebrospinal fluid
GPD: Generalized Pareto distribution
GWAS: Genome-wide association studies
LD: Linkage disequilibrium
MAF: Minor allele frequency
MCI: Cognitive impairment
MRI: Magnetic resonance imaging
PET: Positron emission tomography
QPSS: Quantitative phenotype scan statistic
SKAT: Sequence kernel association test
SNPs: Single nucleotide polymorphisms
SNVs: Single nucleotide variants
WES: Whole-exome sequencing
WGS: Whole-genome sequencing
