# Supplementary material for: Quantitative phenotype scan statistic (QPSS) reveals rare variant associations with Alzheimer’s disease endophenotypes
Source: BMC Med Genet. 2020 May 15;21:106. doi: 10.1186/s12881-020-01046-6 (PMC7229597; doi:10.1186/s12881-020-01046-6)
Supplement: Supplementary file 1 — Additional file 1: Supplementary Method 1. Scan statistics for the normal probability model developed by Kulldorff et al. [1]. Supplementary Method 2. Computing empirical p-values based on permutation test and approximation by a generalized Pareto distribution described by Knijnenburg et al. [2]. Table S1. Simulation scenario for type I error and power evaluations. Table S2. Frequency of the number of targeted sliding windows that produced the maximum value of $\ln {\hat{LR}}_W$lnLR^W (n = 500). Table S3. Frequency of the number of targeted sliding windows that produced the maximum value of $\ln {\hat{LR}}_W$lnLR^W (n = 1000). Table S4. Single variant associations on the significant window of TOMM40 (45,403,046 – 45,405,045) with log-transformed cerebrospinal fluid amyloid β 1–42 levels in ADNI. Table S5. Single variant associations on the significant window of intergenic region (45,412,796 – 45,413,295) with log-transformed cerebrospinal fluid amyloid β 1–42 levels in ADNI. Table S6. Single variant associations on the significant window of ARHGAP23 (36,637,321 – 36,638,320) with log-transformed cerebrospinal fluid phosphorylated tau levels in ADNI. Figure S1. Mean of continuous phenotype y in each scenario. Figure S2. Estimate of heritability in each scenario. Figure S3. Mean of [file 12881_2020_1046_MOESM1_ESM.pdf]

Supplementary Methods

Supplementary Method 1

Scan statistics for the normal probability model developed by Kulldorff et al [1].

Under the null hypothesis, the likelihood function for the normal probability model is

$$L_0 = \prod_i \frac{1}{\sqrt{2\pi\sigma^2}} \exp\left\{-\frac{(y_i - \mu)^2}{2\sigma^2}\right\}$$

Under the alternative, the likelihood function is

$$L_1 = \prod_{i \in W_+} \frac{1}{\sqrt{2\pi\sigma_W^2}} \exp\left\{-\frac{\left(y_i - \mu_{W_+}\right)^2}{2\sigma_W^2}\right\} \prod_{i \in W_-} \frac{1}{\sqrt{2\pi\sigma_W^2}} \exp\left\{-\frac{\left(y_i - \mu_{W_-}\right)^2}{2\sigma_W^2}\right\}$$

where σ_W^2 is the common variance of y_i for the sets W_+ and W_-. That is, we assume that only the mean shifts under the alternative, and the trait variance within subjects carrying a rare variant in window W is the same as the variance of subjects with a rare variant in G but not in W. The log likelihood ratio is

$$\begin{aligned} \ln\frac{L_1}{L_0} &= \ln L_1 - \ln L_0 \\ &= -\frac{n}{2}\ln\sigma_W^2 - n\ln\sqrt{2\pi} - \sum_{i \in W_+} \frac{\left(y_i - \mu_{W_+}\right)^2}{2\sigma_W^2} - \sum_{i \in W_-} \frac{\left(y_i - \mu_{W_-}\right)^2}{2\sigma_W^2} \\ &\quad + \frac{n}{2}\ln\sigma^2 + n\ln\sqrt{2\pi} + \sum_i \frac{(y_i - \mu)^2}{2\sigma^2} \\ &= \frac{n}{2}\ln\frac{\sigma^2}{\sigma_W^2} - \frac{1}{2\sigma_W^2}\left\{\sum_{i \in W_+} \left(y_i - \mu_{W_+}\right)^2 + \sum_{i \in W_-} \left(y_i - \mu_{W_-}\right)^2\right\} + \sum_i \frac{(y_i - \mu)^2}{2\sigma^2} \end{aligned}$$

The maximum likelihood estimate for the variance under the null hypothesis $\sigma^2$is

$$\hat{\sigma}^2 = \frac{1}{n}\sum_i (y_i - \hat{\mu})^2$$

where $\hat{\mu}$ is the maximum likelihood estimate of mean under the null hypothesis. The maximum likelihood estimate for the common variance σ_W^2 is

$$\hat{\sigma}_W^2 = \frac{1}{n}\left\{\sum_{i \in W_+} \left(y_i - \hat{\mu}_{W_+}\right)^2 + \sum_{i \in W_-} \left(y_i - \hat{\mu}_{W_-}\right)^2\right\}$$

where $\hat{\mu}_{W_+}$ and $\hat{\mu}_{W_-}$ are the maximum likelihood estimates of means inside and outside the window, respectively. Therefore the log likelihood ratio test statistic is expressed as

$$\ln\widehat{LR}_W = \frac{n}{2}\ln\frac{\hat{\sigma}^2}{\hat{\sigma}_W^2}$$

Supplementary Method 2

Computing empirical p-values based on permutation test and approximation by a generalized Pareto distribution described by Knijnenburg et al [2]

Permutation tests are non-parametric procedures and the p-values are obtained by comparing a test statistic with permutation values as

$$p_{prem} = \frac{1 + \sum_{i=1}^{m} I(x_i \geq x_{ts})}{m+1}$$

where x_{ts} is the test statistic, x_i is the ith permutation value ($i = 1, \dots, m$), and $I(.)$ is the indicator function. A pseudocount of 1 is added to avoid p-values of zero [3]. However, the permutation p-value should be computed by performing all m possible distinct permutaions, which is computationally expensive or even unfesible in practice. We applied a method with less computational burden that Knijnenburg et al. proposed to estimate the small permutation p-values [2]. It assumes that the tail of the distribtuion of permutation values follows a generalized Pareto distribution (GPD), which cumulative distirbution function is expressed as

$$F(z) = \begin{cases} 1 - \left(1 - \frac{kz}{a}\right)^{\frac{1}{k}}, & k \neq 0 \\ 1 - e^{-\frac{z}{a}}, & k = 0 \end{cases}$$

where is the scale parameter, k is the shape parameter, the range of z is $0 \leq z < \infty$ for $k \leq 0$ and is $0 \leq z \leq a/k$ for $k > 0$. The permutation p-value with GPD approximation is compuated as

$$p_{GPD} = \frac{m_{exc}}{m^*}\left(1 - F(x_{ts} - t)\right)$$

where m^* is the number of permutation values (usually $m^* \ll m$) and t is the threshold for GPD. As Knijnenburg et al. proposed, we chose m_{exc}= 250 and set t as $\left(x^*_{m_{exc}} + x^*_{m_{exc+1}}\right)/2$, where x_i^* is the ith ordered permutaion value such that $x_1^* \geq x_2^* \geq \cdots \geq x_m^*$. We performed the goodness-of-fit test using “gp_test” function in the “goft” R pacakge [4] for the permutation

values. If the 250 permutation values do not follow a GPD according to the test, the m_{exc} is decreased until the p-value < 0.05 of goodness-of-fit test.

We limited the number of permutation steps to $m^{*} = 10^{5}$. If $\sum_{i=1}^{m^{*}} I(x_i \geq x_{ts}) \geq 100$, we computed p_{prem}, otherwise we computed p_{GPD}. Using the method of moment, we estimated two parameters a and k as

$$\hat{a} = \frac{\bar{z}}{2}\left(\frac{\bar{z}^2}{s^2} + 1\right), \quad \hat{k} = \frac{1}{2}\left(\frac{\bar{z}^2}{s^2} - 1\right)$$

where $\bar{z}$ and s^2 are the mean and variance of the values z, respectively.

References

1. Kulldorff, M., L. Huang, and K. Konty, *A scan statistic for continuous data based on the normal probability model.* Int J Health Geogr, 2009. **8**: p. 58.
2. Knijnenburg, T.A., et al., *Fewer permutations, more accurate P-values.* Bioinformatics, 2009. **25**(12): p. i161-8.
3. Phipson, B. and G.K. Smyth, *Permutation P-values should never be zero: calculating exact P-values when permutations are randomly drawn.* Stat Appl Genet Mol Biol, 2010. **9**(1): p. Article39.
4. González-Estrada, E. and J.A. Villaseñor, *An R package for testing goodness of fit: goft.* Journal of Statistical Computation and Simulation, 2018. **88**(4): p. 726-751.

Supplementary Tables

Supplementary Table 1. Simulation scenario for type I error and power evaluations

Cluster size (bp)	Start position	End position	# of variants m_C	# of independent variants
200	828,704	828,703	4	4
500	658,914	659,413	10	9
2 k	351,925	353,924	8	7

Supplementary Table 2. Frequency of the number of targeted sliding windows that produced the maximum value of $\ln\widehat{LR}_W$ (n = 500)

Cluster	Window size	Targeted window position [a]	# of windows		
			c = 0.2	c = 0.4	c = 0.6
Cluster size = 200 bp	5k	825,071 – 830,070	4	3	9
		827,571 – 832,570	4	3	5
Cluster position = 828,704 – 828,703	2k	827,071 – 829,070	1	16	19
		828,071 – 830,070	5	19	41
	1k	828,071 – 829,070	7	34	94
# of variants (m_C) = 4		828,571 – 829,570	3	54	181
(4 independent variants)	500	828,321 – 828,820	0	10	7
		828,571 – 829,070	11	159	540
		828,821 – 829,320	0	0	0
Cluster size = 500 bp	5k	655,071 – 660,070	40	218	488
		657,571 – 662,570	18	59	88
Cluster position	2k	657,071 – 659,070	32	17	3
= 658,914 – 659,413		658,071 – 660,070	196	844	988
		659,071 – 661,070	14	12	0
# of variants (m_C) = 10	1k	658,071 – 659,070	43	61	23
(9 independent variants)		658,571 – 659,570	184	811	965
		659,071 – 660,070	32	23	5
	500	658,571 – 659,070	21	10	1
		658,821 – 659,320	249	804	962
		659,071 – 659,570	58	72	24
		659,321 – 659,820	4	0	0
Cluster size = 2 kbp	5k	347,571 – 352,570	4	7	5
(containing 20% disease-		350,071 – 355,070	19	29	40
related variants)		352,571 – 357,570	1	0	0
	2k	350,071 – 352,070	11	24	15
Cluster position		351,071 – 353,070	18	58	52
= 351,925 – 353,924		352,071 – 354,070	59	487	861
		353,071 – 355,070	8	0	0
# of variants (m_C) = 8	1k	351,071 – 352,070	7	10	3
(7 independent variants)		351,571 – 352,570	31	101	39
		352,071 – 353,070	112	492	758
		352,571 – 353,570	53	189	147
		353,071 – 354,070	8	5	1
		353,571 – 354,570	4	1	0
	500	351,571 – 352,070	31	89	55
		351,821 – 352,320	0	0	0
		352,071 – 352,570	1	1	0
		352,321 – 352,820	1	0	0
		352,571 – 353,070	45	175	224
		352,821 – 353,320	23	27	9
		353,071 – 353,570	43	132	189
		353,321 – 353,820	18	58	37
		353,571 – 354,070	0	1	0
		353,821 – 354,320	0	0	0

[a] Window that overlaps any of the true risk variant cluster

Supplementary Table 3. Frequency of the number of targeted sliding windows that produced the maximum value of $\ln\widehat{LR}_W$ (n = 1,000)

Cluster	Window size	Targeted window position [a]	# of windows c = 0.2	# of windows c = 0.4	# of windows c = 0.6
Cluster size = 200 bp	5k	825,071 – 830,070	8	10	17
		827,571 – 832,570	10	11	13
Cluster position	2k	827,071 – 829,070	5	22	28
= 828,704 – 828,703		828,071 – 830,070	7	28	80
	1k	828,071 – 829,070	9	68	179
# of variants (m_C) = 4		828,571 – 829,570	17	147	441
(4 independent variants)	500	828,321 – 828,820	6	11	5
		828,571 – 829,070	61	554	948
		828,821 – 829,320	1	0	0
Cluster size = 500 bp	5k	655,071 – 660,070	82	429	763
		657,571 – 662,570	29	77	41
Cluster position	2k	657,071 – 659,070	47	11	0
= 658,914 – 659,413		658,071 – 660,070	462	979	1000
		659,071 – 661,070	10	1	0
# of variants (m_C) = 10	1k	658,071 – 659,070	73	15	0
(9 independent variants)		658,571 – 659,570	469	974	1000
		659,071 – 660,070	30	1	0
	500	658,571 – 659,070	28	2	0
		658,821 – 659,320	503	913	991
		659,071 – 659,570	76	41	5
		659,321 – 659,820	1	0	0
Cluster size = 2 kbp	5k	347,571 – 352,570	4	0	0
(containing 20% disease-		350,071 – 355,070	28	52	82
related variants)		352,571 – 357,570	4	5	0
	2k	350,071 – 352,070	12	4	0
Cluster position		351,071 – 353,070	34	27	5
= 351,925 – 353,924		352,071 – 354,070	237	884	992
		353,071 – 355,070	1	0	0
# of variants (m_C) = 8	1k	351,071 – 352,070	7	0	0
(7 independent variants)		351,571 – 352,570	76	35	3
		352,071 – 353,070	319	793	914
		352,571 – 353,570	146	160	83
		353,071 – 354,070	26	2	0
		353,571 – 354,570	3	0	0
	500	351,571 – 352,070	101	107	55
		351,821 – 352,320	0	0	0
		352,071 – 352,570	2	0	0
		352,321 – 352,820	0	0	0
		352,571 – 353,070	233	620	793
		352,821 – 353,320	31	3	1
		353,071 – 353,570	48	58	21
		353,321 – 353,820	94	105	76
		353,571 – 354,070	5	0	0
		353,821 – 354,320	0	0	0

[a] Window that overlaps any of the true risk variant cluster

Supplementary Table 4. Single variant associations on the significant window of *TOMM40* (45,403,046 – 45,405,045) with log-transformed cerebrospinal fluid amyloid β 1-42 levels in ADNI

rs ID	Position [a]	MAF	Genotype	n	Log-transformed CSF amyloid β 1-42 levels		
					Mean	SD	P-value [b]
rs417357	45,403,119	0.0028	T/C	3	4.749	0.154	0.028
			C/C	533	5.143	0.309	
rs115881343	45,403,216	0.046	T/T	1	4.615	-	4.3×10^{-9}
			T/C	47	4.901	0.255	
			C/C	490	5.166	0.304	
rs77100236	45,403,458	0.0046	T/C	5	5.082	0.329	0.66
			C/C	533	5.143	0.310	
rs567994175	45,403,511	0.0019	A/G	2	5.095	0.322	0.83
			G/G	536	5.142	0.310	
rs142412517	45,404,058	0.0019	T/C	2	4.741	0.399	0.067
			C/C	536	5.144	0.309	
rs73052321	45,404,121	0.011	A/G	12	5.025	0.307	0.19
			G/G	526	5.145	0.310	
rs117264457	45,404,432	0.014	A/G	15	4.987	0.283	0.049
			G/G	523	5.147	0.310	
rs394819	45,404,579	0.0028	T/G	3	4.749	0.154	0.027
			G/G	535	5.144	0.309	
rs116977783 [c]	45,404,721	0.013	T/C	14	5.156	0.326	0.87
			C/C	524	5.142	0.310	
rs112019714	45,404,857	0.046	C/C	1	4.615	-	2.6×10^{-9}
			C/T	48	4.900	0.252	
			T/T	489	5.167	0.304	
rs183743534 [c]	45,404,866	0.0019	A/G	2	5.340	0.0237	0.37
			G/G	536	5.141	0.310	
rs144738835	45,404,926	0.0019	C/G	2	4.671	0.105	0.031
			G/G	535	5.143	0.309	
rs534150972	45,404,963	0.0047	G/C	5	5.054	0.397	0.52
			C/C	532	5.143	0.309	

[a] Chromosomal positions are based on GRCh37/hg19 human genome assembly.
[b] P-values were computed assuming dominant mode of inheritance.
[c] Positive association with CSF amyloid β 1-42.
MAF = minor allele frequency; CSF = cerebrospinal fluid; SD = standard deviation

Supplementary Table 5. Single variant associations on the significant window of intergenic region (45,412,796 – 45,413,295) with log-transformed cerebrospinal fluid amyloid β 1-42 levels in ADNI

rs ID	Position [a]	MAF	Genotype	n	Log-transformed CSF amyloid β 1-42 levels		
					Mean	SD	P-value [b]
rs1081105	45,412,955	0.046	C/C	1	4.615	-	4.5×10^{-9}
			C/A	47	4.901	0.255	
			A/A	489	5.166	0.304	
rs186363642	45,413,207	0.0019	A/T	2	5.015	0.0938	0.57
			T/T	526	5.141	0.312	

[a] Chromosomal positions are based on GRCh37/hg19 human genome assembly.
[b] P-value was computed assuming dominant mode of inheritance.
MAF = minor allele frequency; CSF = cerebrospinal fluid; SD = standard deviation

Supplementary Table 6. Single variant associations on the significant window of *ARHGAP23* (36,637,321 – 36,638,320) with log-transformed cerebrospinal fluid phosphorylated tau levels in ADNI

rs ID	Position [a]	MAF	Genotype [b]	n	Log-transformed CSF phosphorylated tau levels		
					Mean	SD	P-value [c]
rs551160952	36,637,395	0.010	A/T	9	4.012	0.508	0.0029
			T/T	526	3.482	0.528	
rs534336323	36,637,841	0.0019	A/T	2	4.014	0.00383	0.16
			T/T	536	3.490	0.531	
rs144293375	36,638,178	0.013	T/C	17	4.038	0.503	1.4×10^{-5}
			C/C	520	3.473	0.523	

[a] Chromosomal positions are based on GRCh37/hg19 human genome assembly.
[b] There were no subjects with minor allele homozygosity.
[c] P-value was computed assuming dominant mode of inheritance.
MAF = minor allele frequency; CSF = cerebrospinal fluid; SD = standard deviation

Supplementary Figures

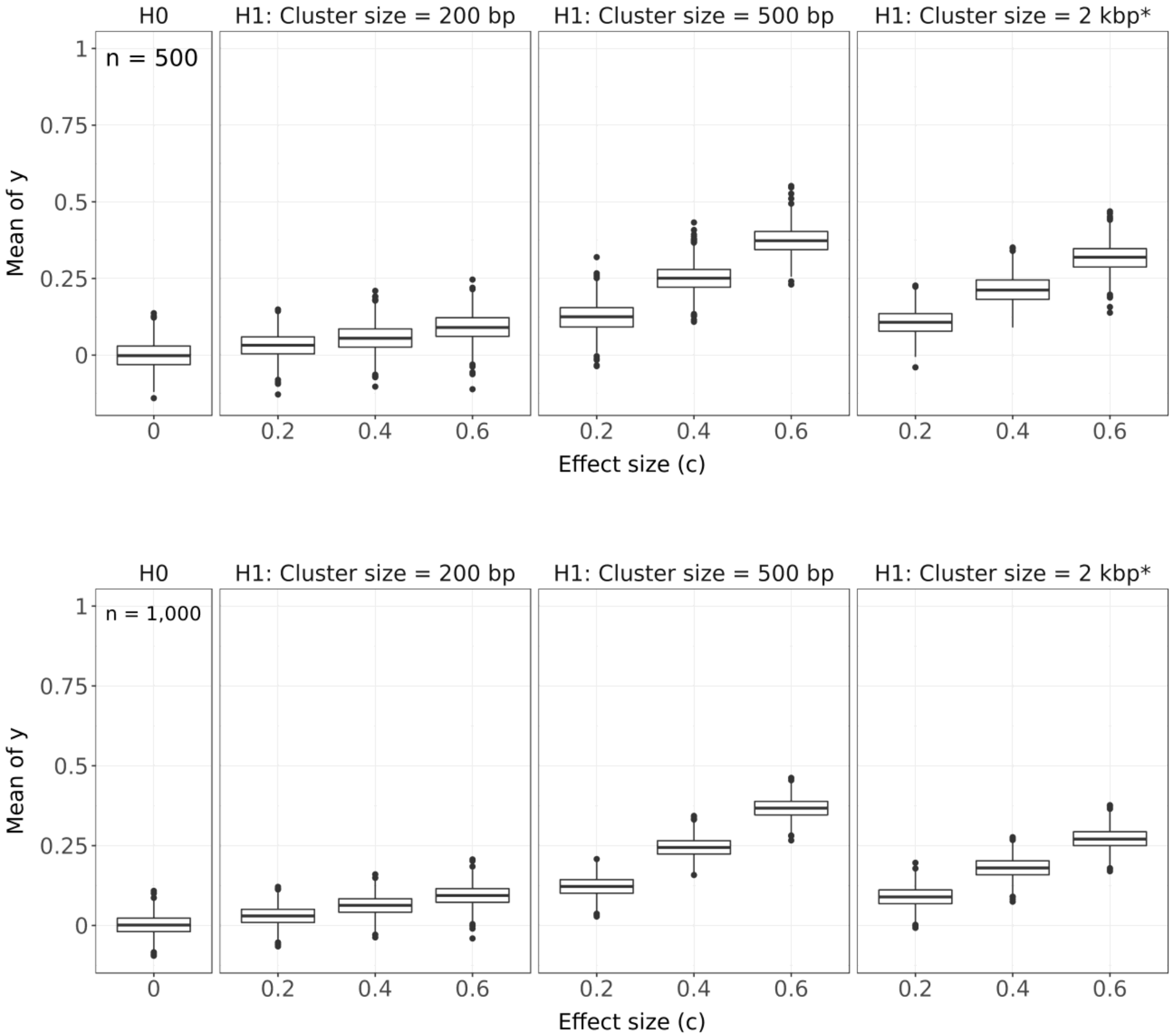

Supplementary Figure 1. Mean of continuous phenotype y in each scenario
[*] Cluster size = 2 kbp contains 20% disease-related variants.

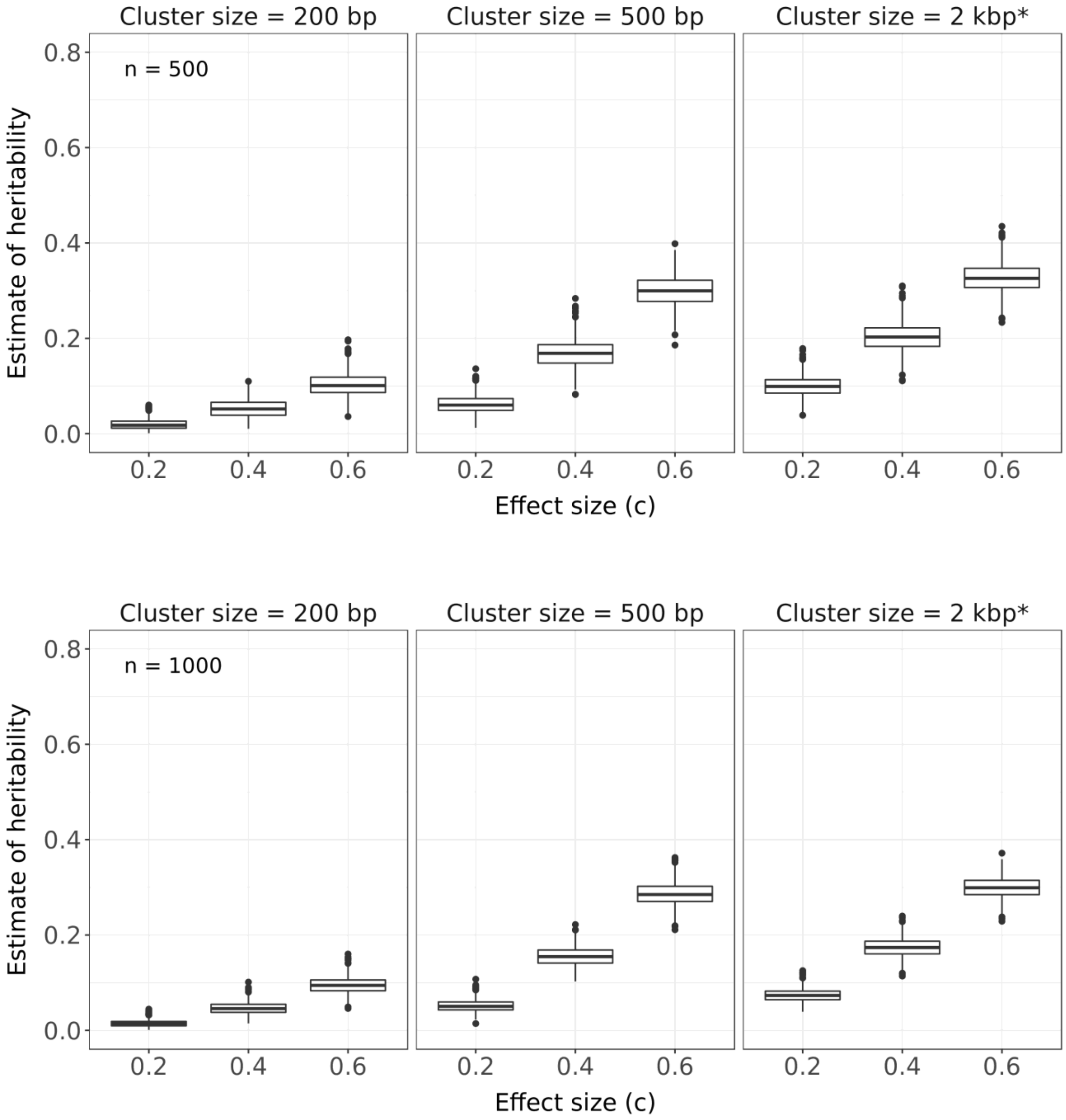

Supplementary Figure 2. Estimate of heritability in each scenario
* Cluster size = 2 kbp contains 20% disease-related variants.

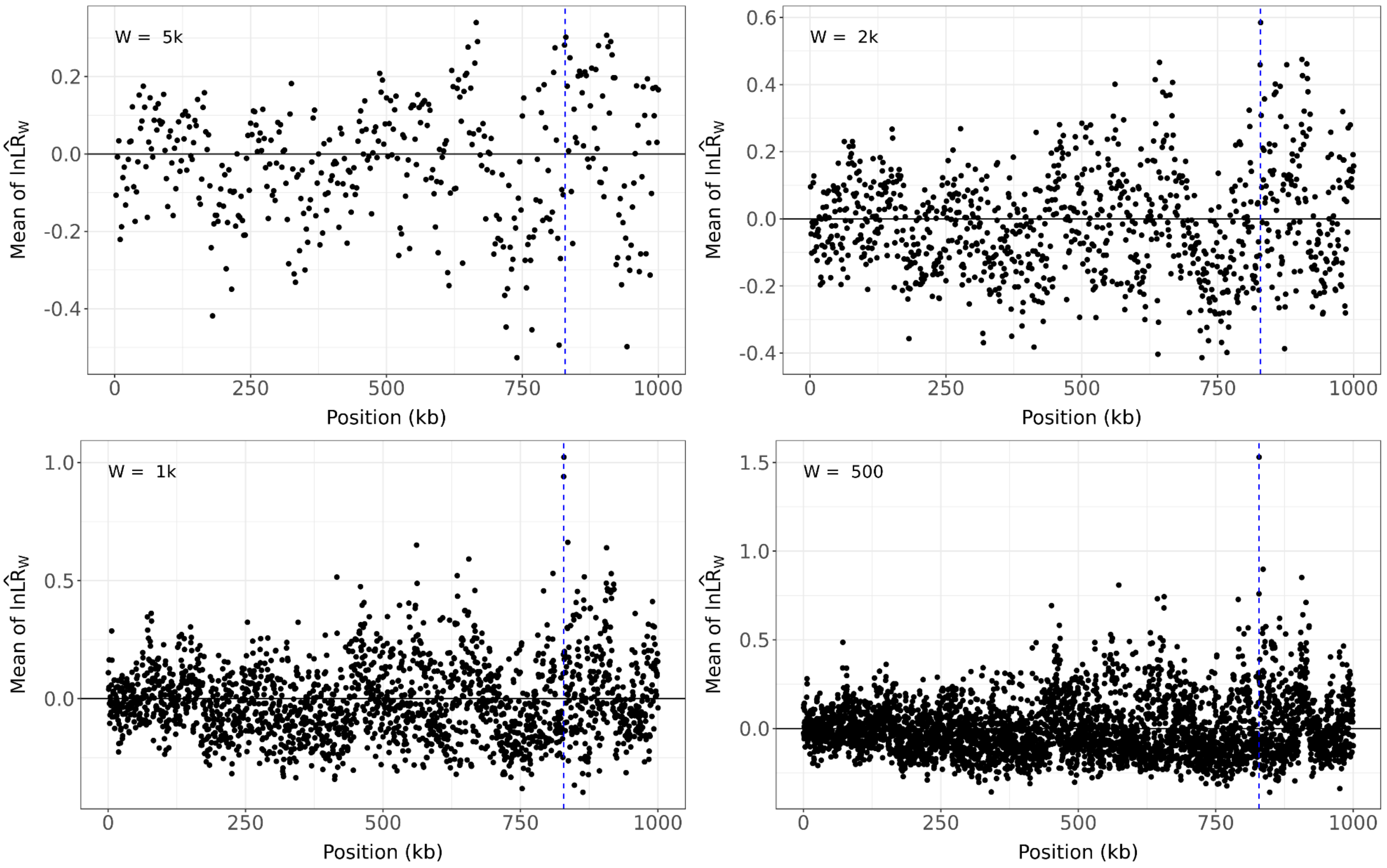

Supplementary Figure 3. Mean of $\ln\widehat{LR}_W$ for n = 500, cluster size = 200 bp, and effect size c = 0.2
Each point represents the center position of each of the windows, and the blue vertical line indicates the center of the cluster position

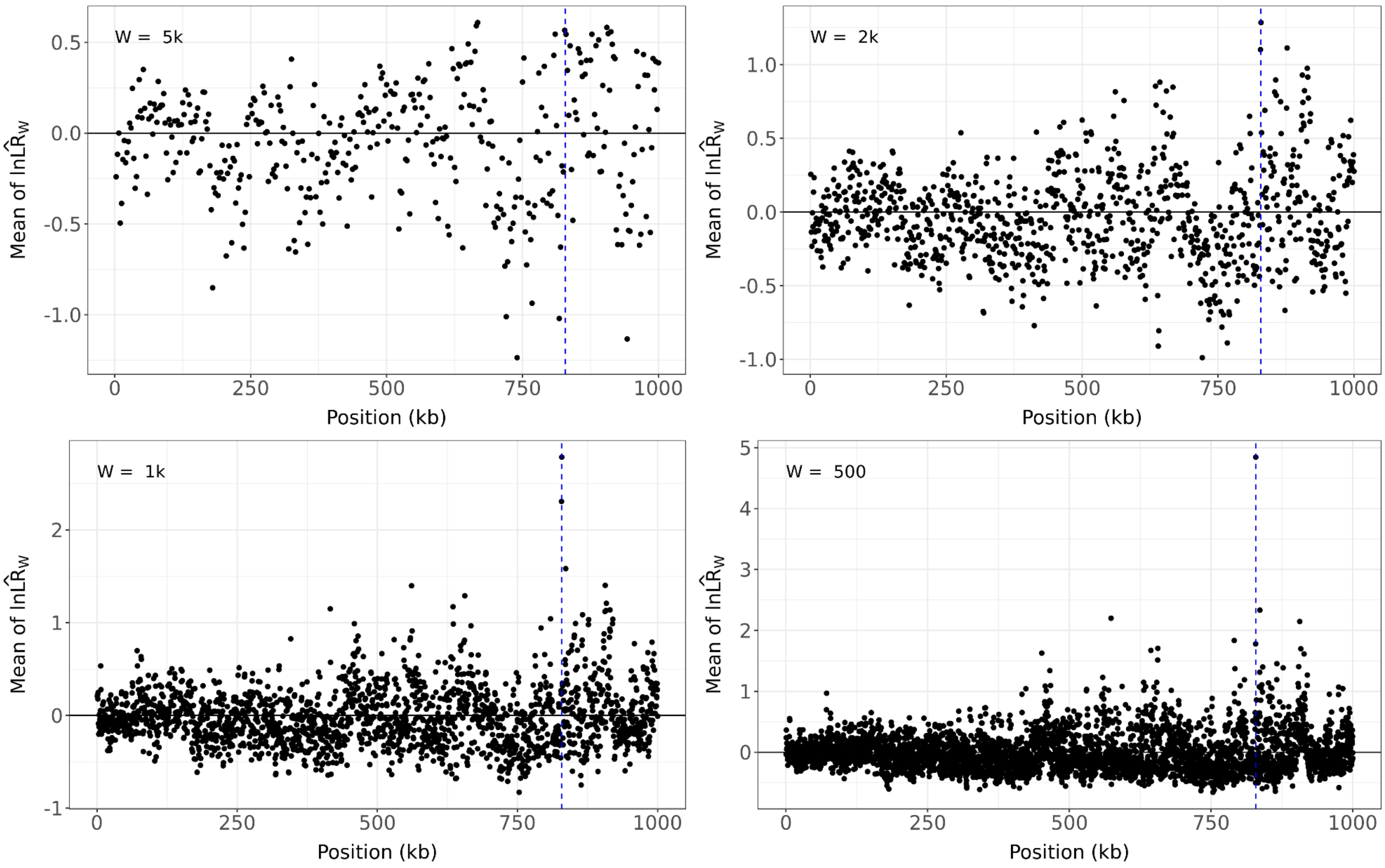

Supplementary Figure 4. Mean of $\ln\widehat{LR}_W$ for n = 500, cluster size = 200 bp, and effect size c = 0.4
Each point represents the center position of each of the windows, and the blue vertical line indicates the center of the cluster position

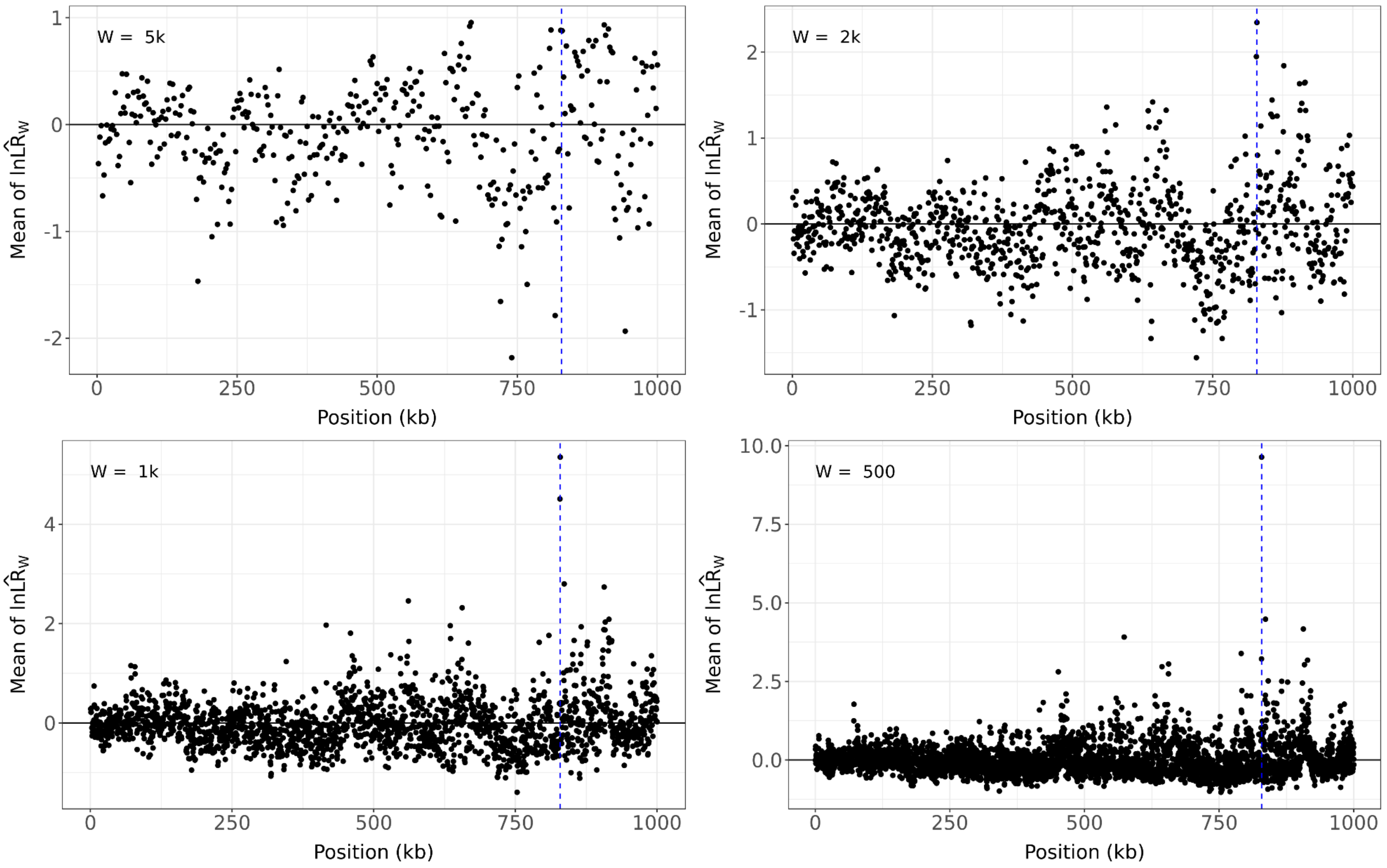

Supplementary Figure 5. Mean of $\ln\widehat{LR}_W$ for n = 500, cluster size = 200 bp, and effect size c = 0.6
Each point represents the center position of each of the windows, and the blue vertical line indicates the center of the cluster position

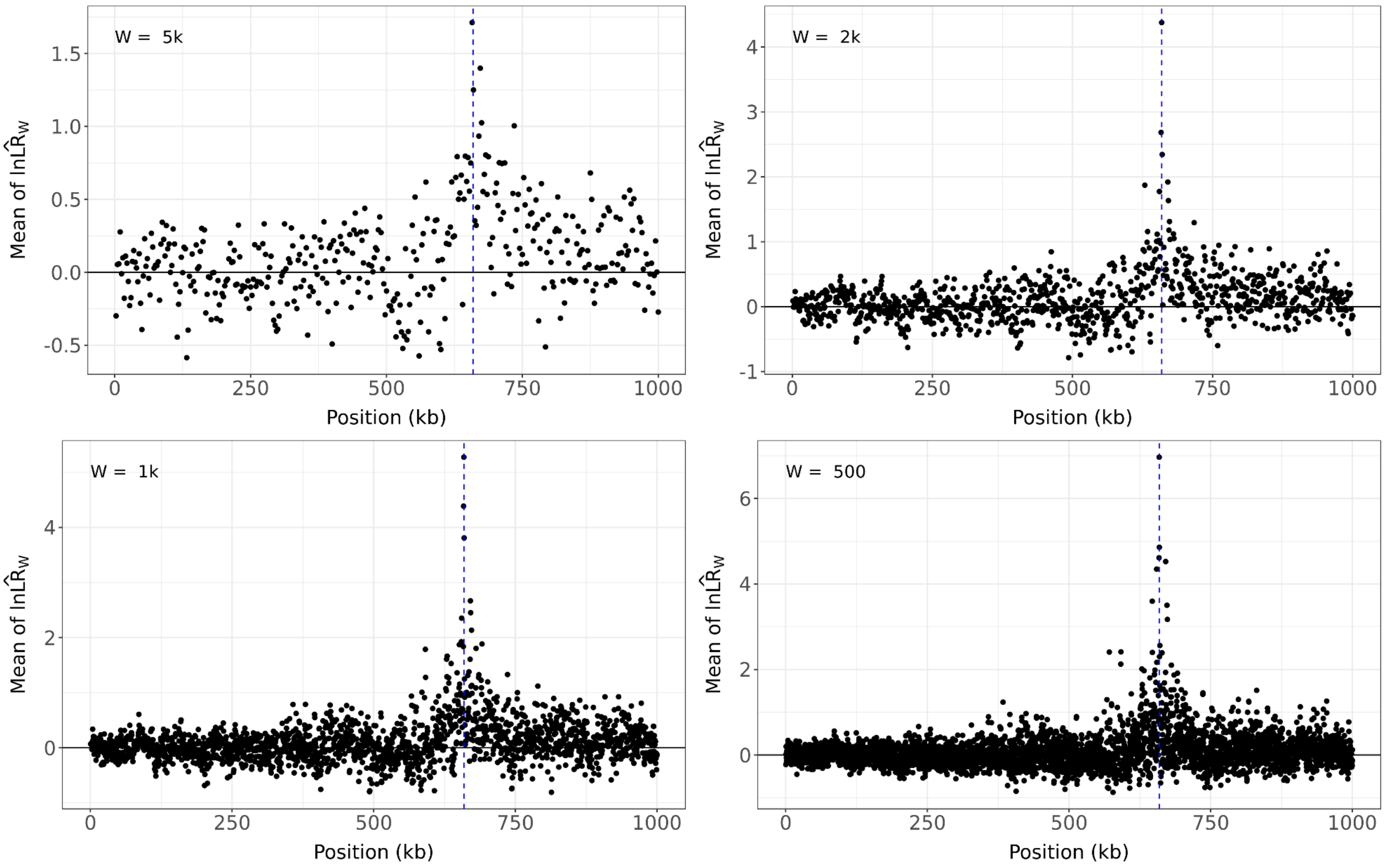

Supplementary Figure 6. Mean of $\ln\widehat{LR}_W$ for n = 500, cluster size = 500 bp, and effect size c = 0.2
Each point represents the center position of each of the windows, and the blue vertical line indicates the center of the cluster position.

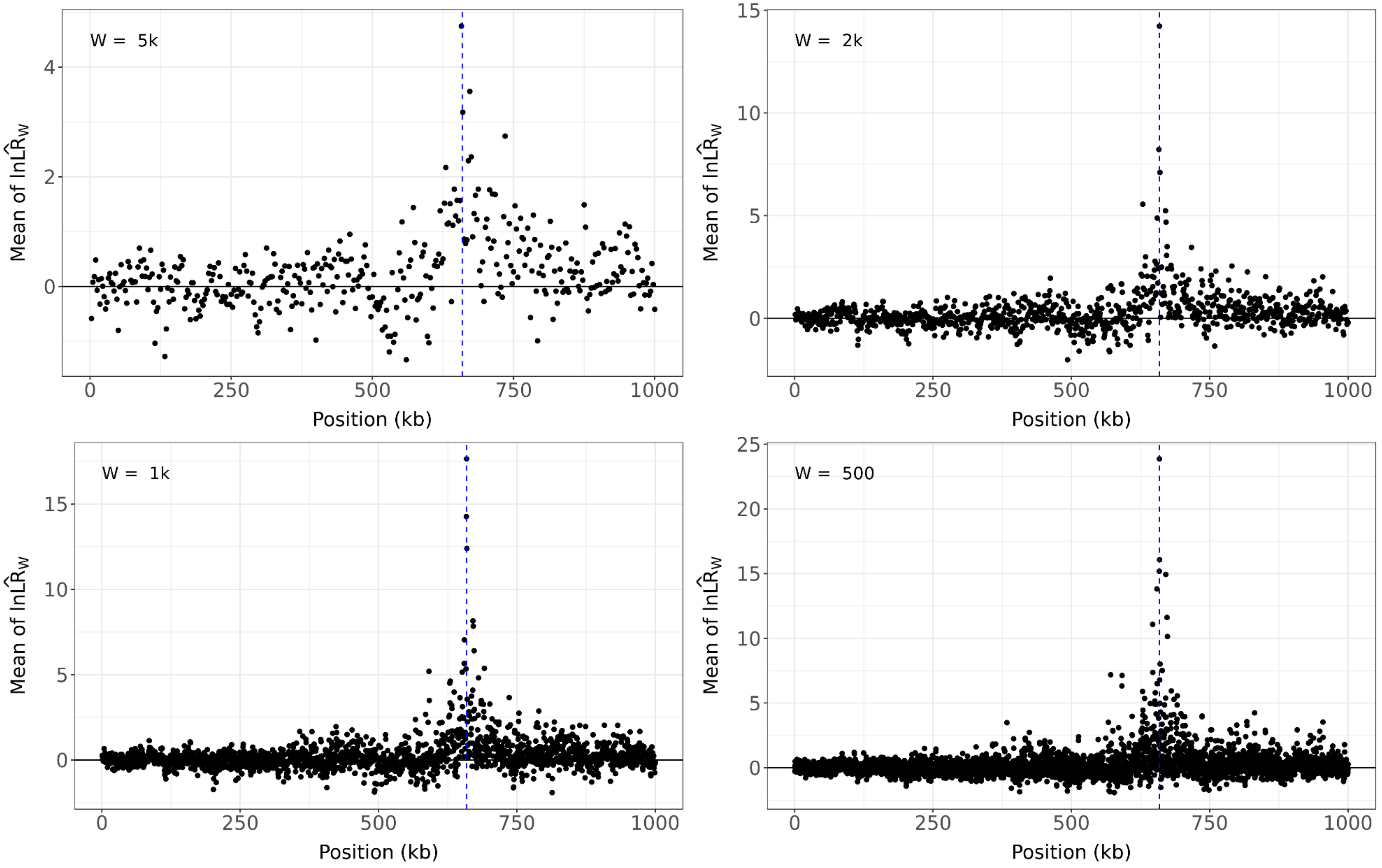

Supplementary Figure 7. Mean of $\ln\widehat{LR}_W$ for n = 500, cluster size = 500 bp, and effect size c = 0.4
Each point represents the center position of each of the windows, and the blue vertical line indicates the center of the cluster position.

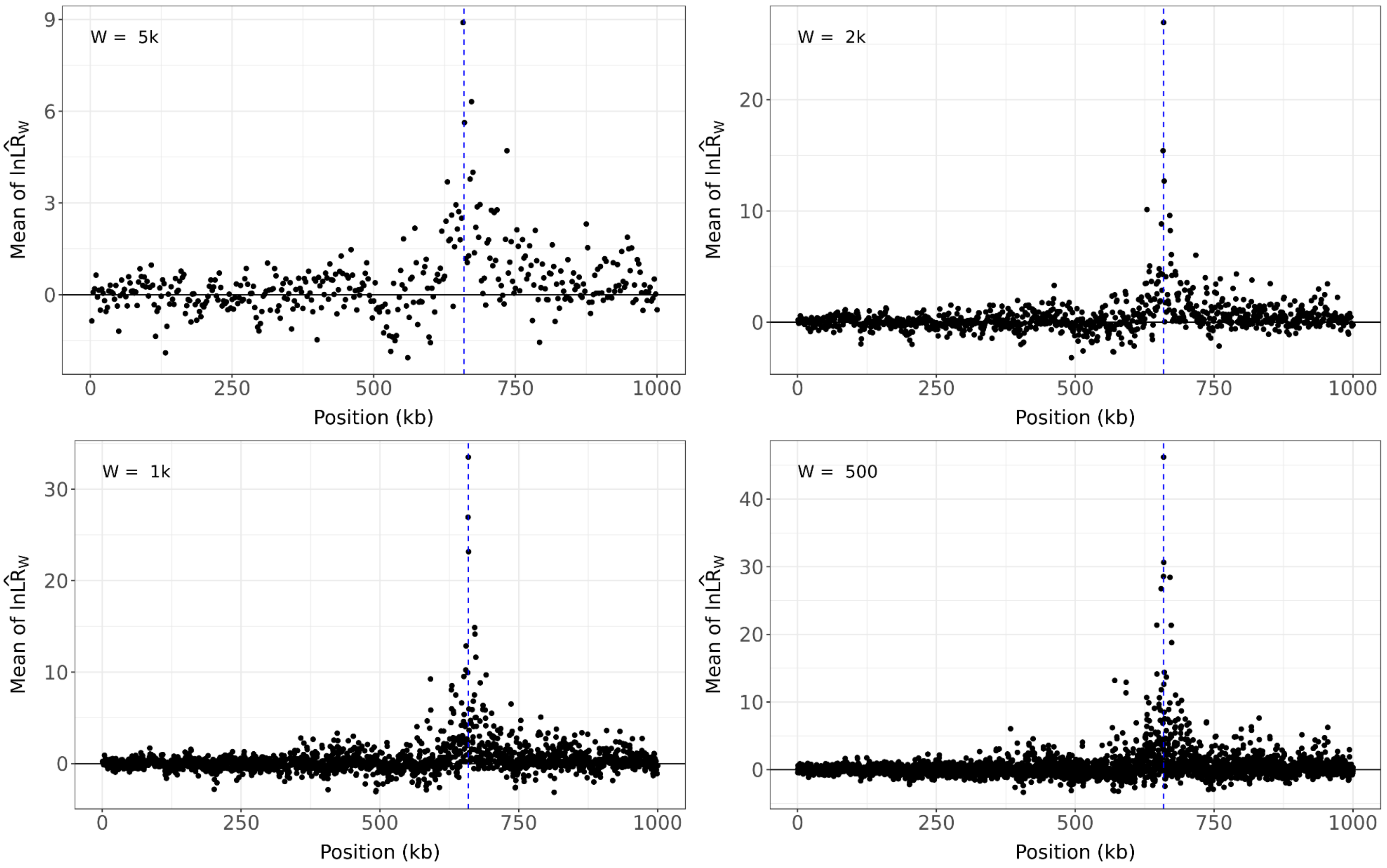

Supplementary Figure 8. Mean of $\ln\widehat{LR}_W$ for n = 500, cluster size = 500 bp, and effect size c = 0.6
Each point represents the center position of each of the windows, and the blue vertical line indicates the center of the cluster position.

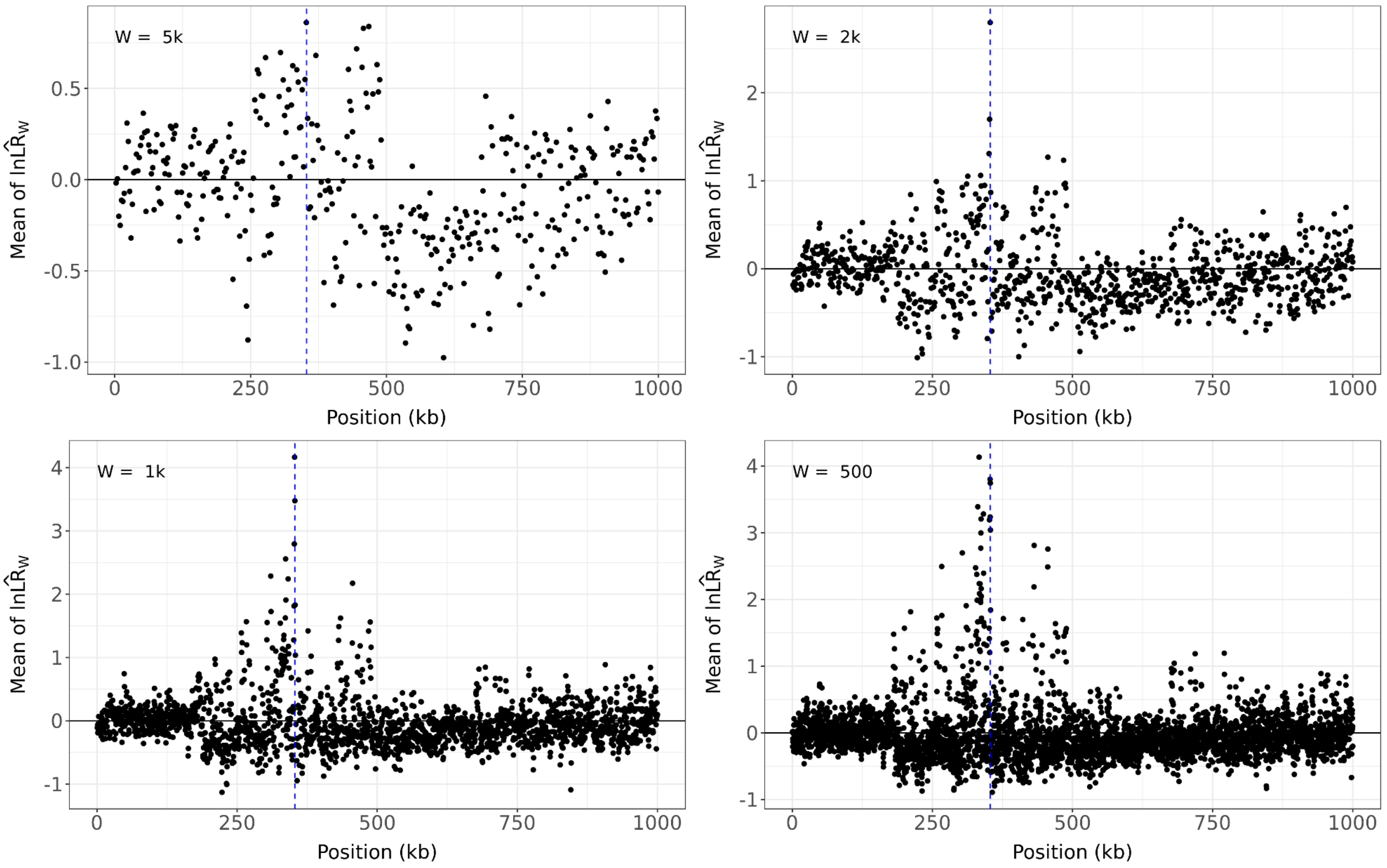

Supplementary Figure 9. Mean of $\ln\widehat{LR}_W$ for n = 500, cluster size = 2 kbp (containing 20% disease-related variants), and effect size c = 0.2

Each point represents the center position of each of the windows, and the blue vertical line indicates the center of the cluster position.

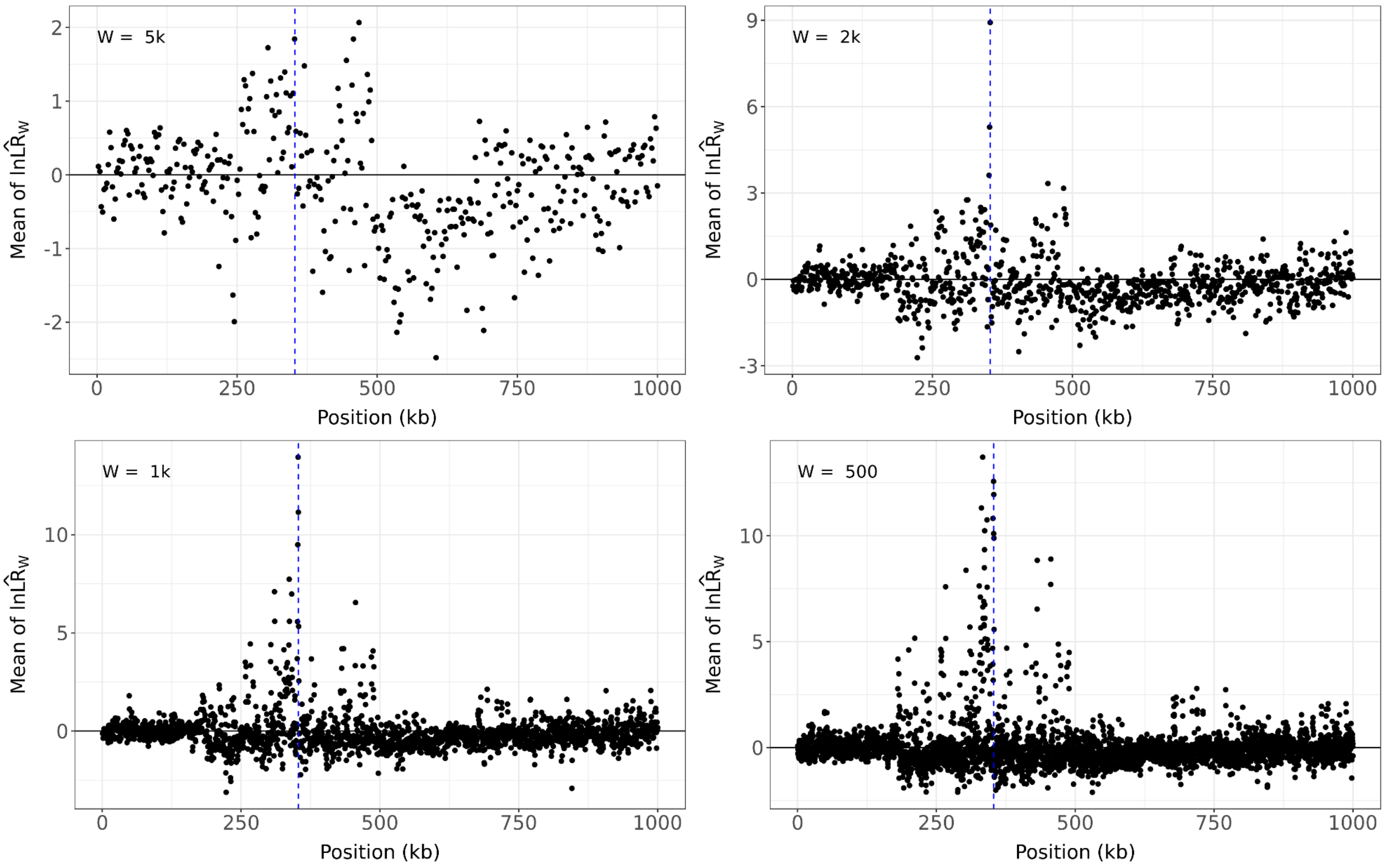

Supplementary Figure 10. Mean of $\ln\widehat{LR}_W$ for n = 500, cluster size = 2 kbp (containing 20% disease-related variants), and effect size c = 0.4

Each point represents the center position of each of the windows, and the blue vertical line indicates the center of the cluster position.

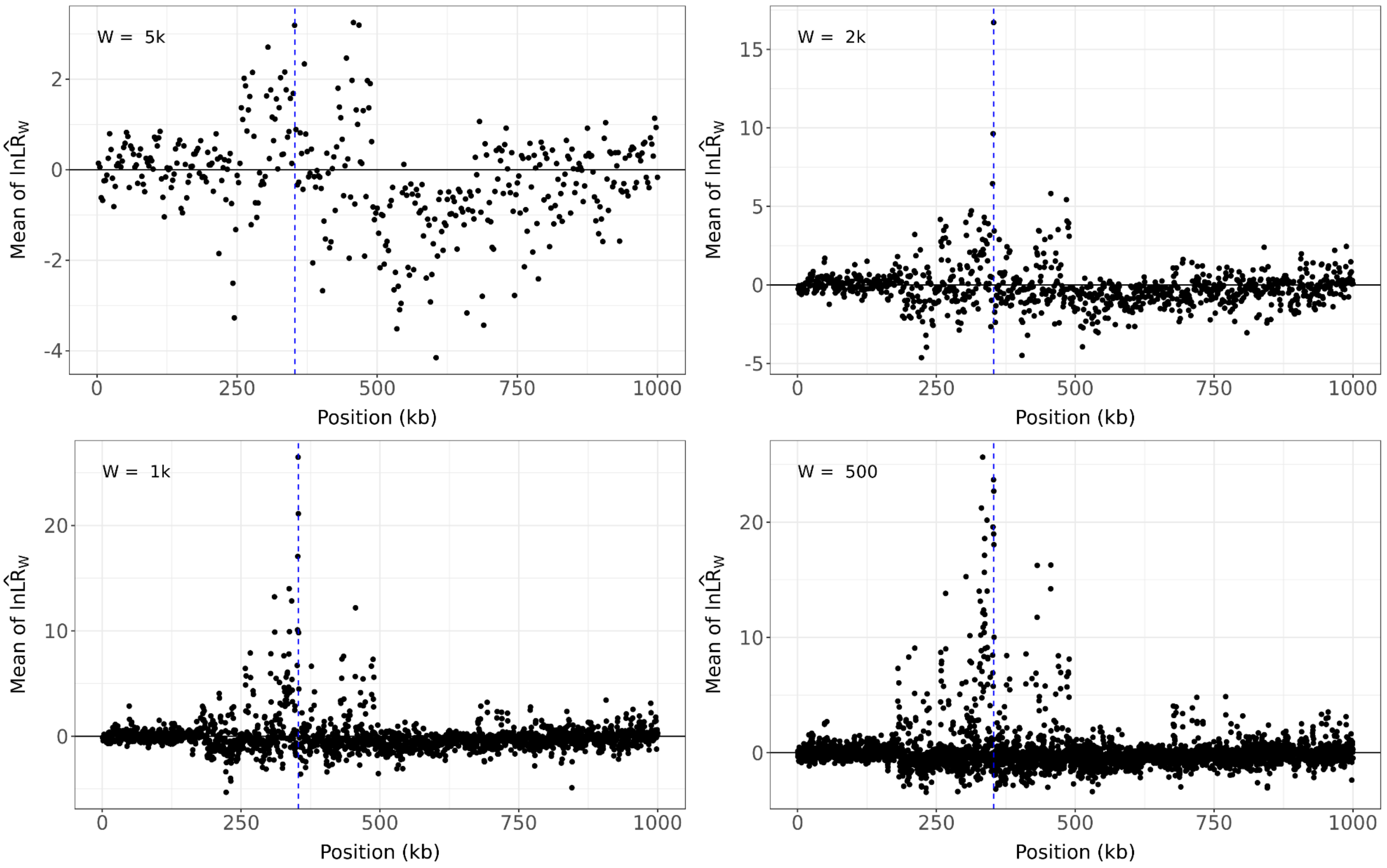

Supplementary Figure 11. Mean of $\ln\widehat{LR}_W$ for n = 500, cluster size = 2 kbp (containing 20% disease-related variants), and effect size c = 0.6

Each point represents the center position of each of the windows, and the blue vertical line indicates the center of the cluster position.

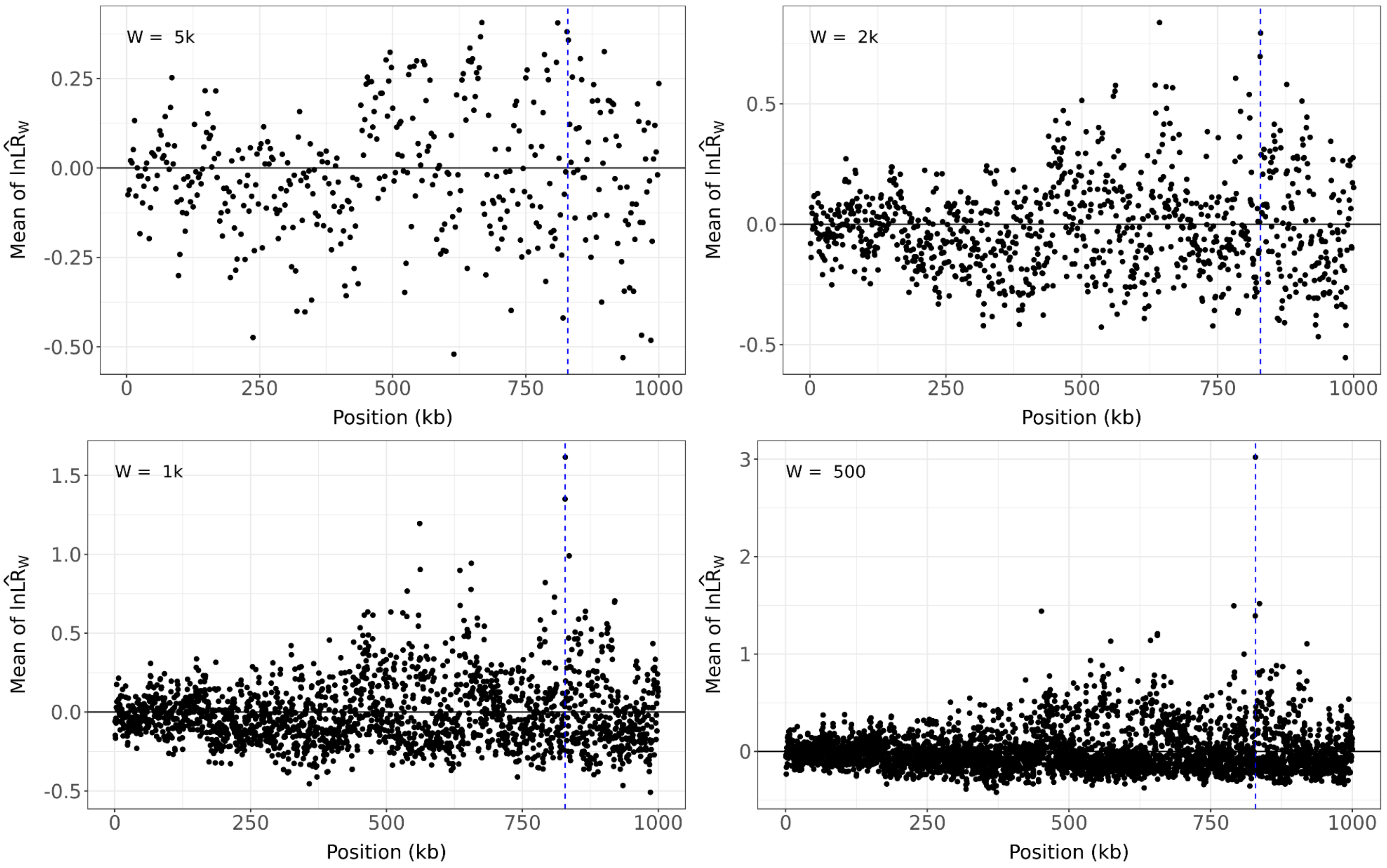

Supplementary Figure 12. Mean of $\ln\widehat{LR}_W$ for n = 1,000, cluster size = 200 bp, and effect size c = 0.2
Each point represents the center position of each of the windows, and the blue vertical line indicates the center of the cluster position

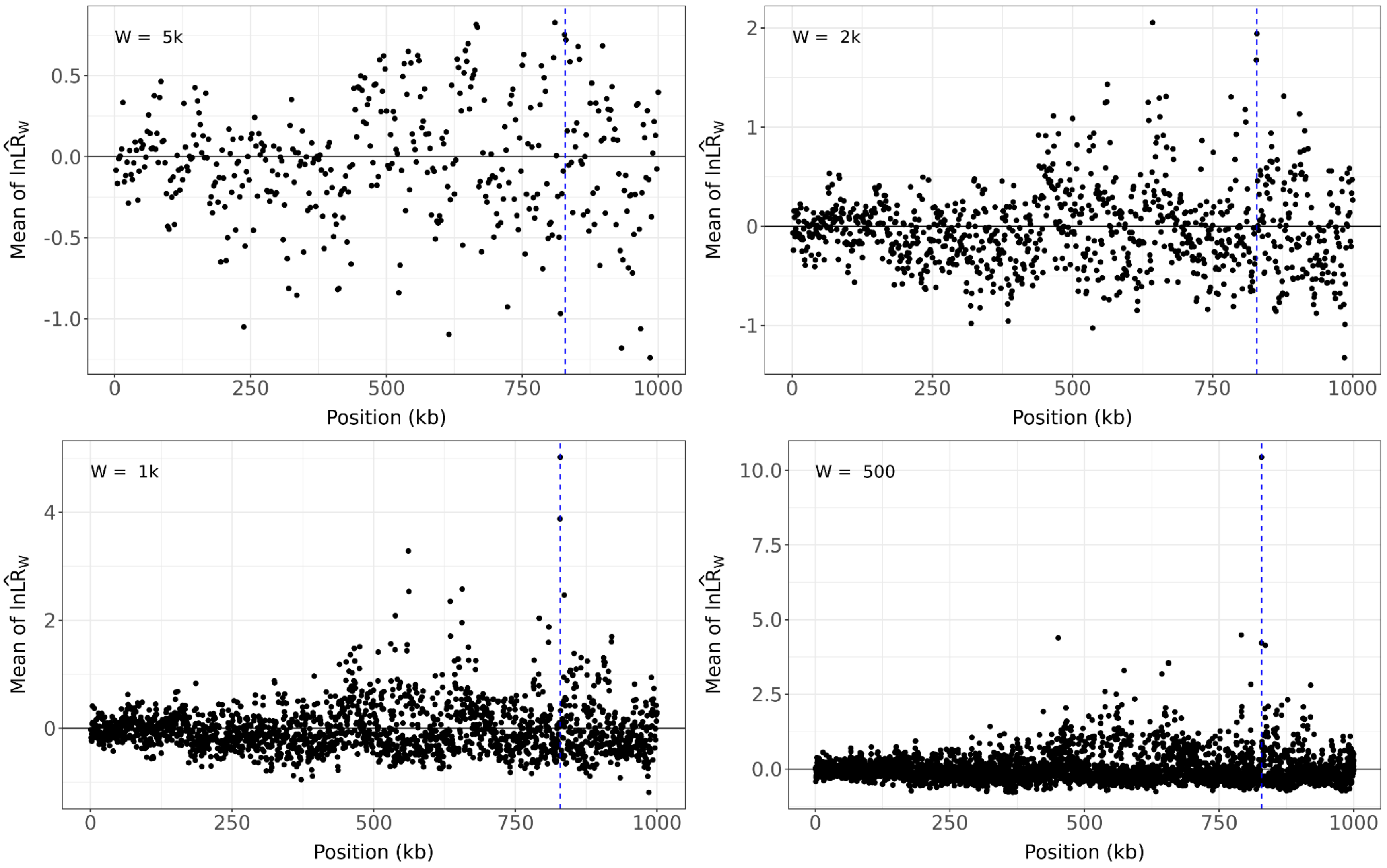

Supplementary Figure 13. Mean of $\ln\widehat{LR}_W$ for n = 1,000, cluster size = 200 bp, and effect size c = 0.4
Each point represents the center position of each of the windows, and the blue vertical line indicates the center of the cluster position

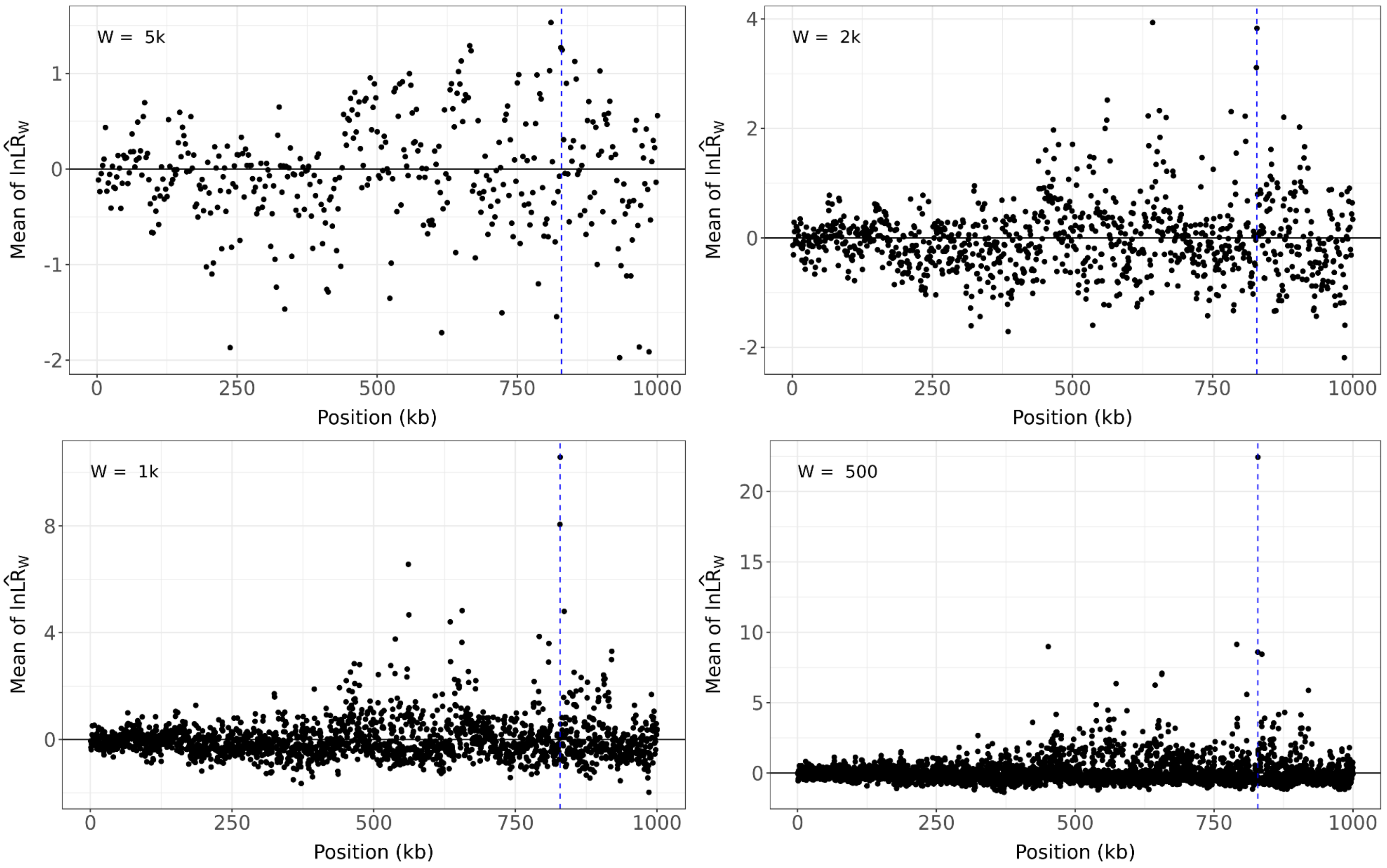

Supplementary Figure 14. Mean of $\ln\widehat{LR}_W$ for n = 1,000, cluster size = 200 bp, and effect size c = 0.6
Each point represents the center position of each of the windows, and the blue vertical line indicates the center of the cluster position

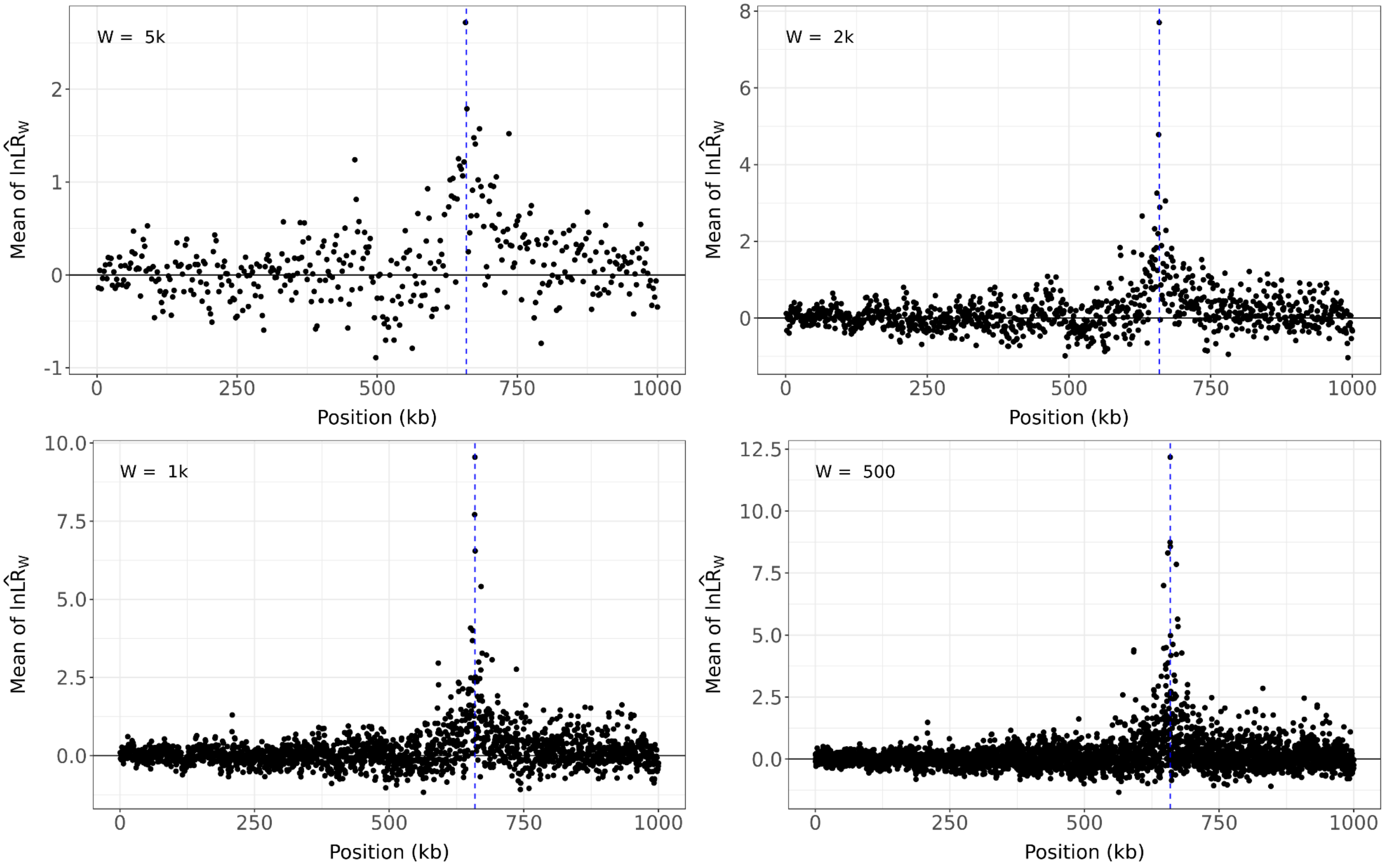

Supplementary Figure 15. Mean of $\ln\widehat{LR}_W$ for n = 1,000, cluster size = 500 bp, and effect size c = 0.2
Each point represents the center position of each of the windows, and the blue vertical line indicates the center of the cluster position.

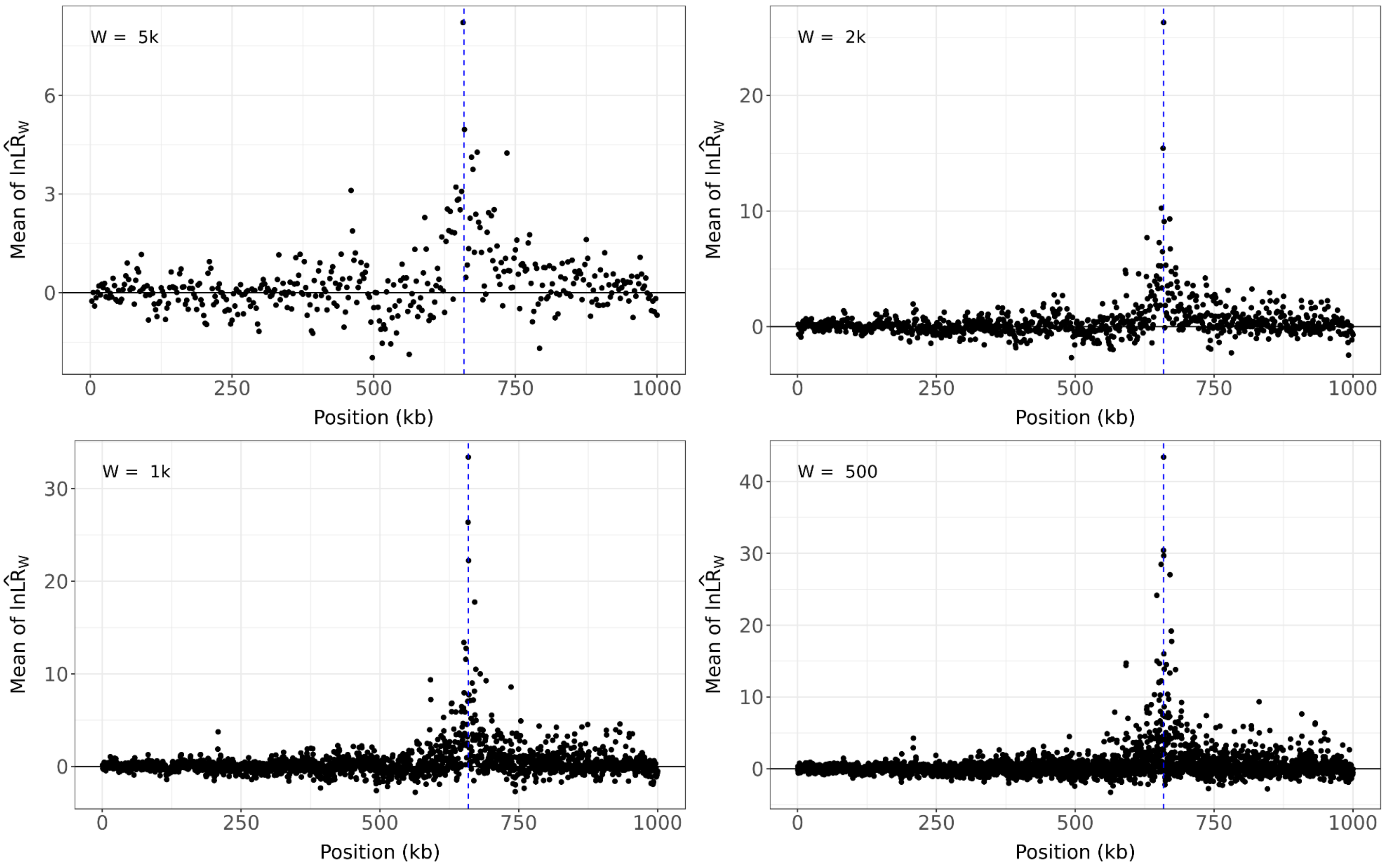

Supplementary Figure 16. Mean of $\ln\widehat{LR}_W$ for n = 1,000, cluster size = 500 bp, and effect size c = 0.4
Each point represents the center position of each of the windows, and the blue vertical line indicates the center of the cluster position.

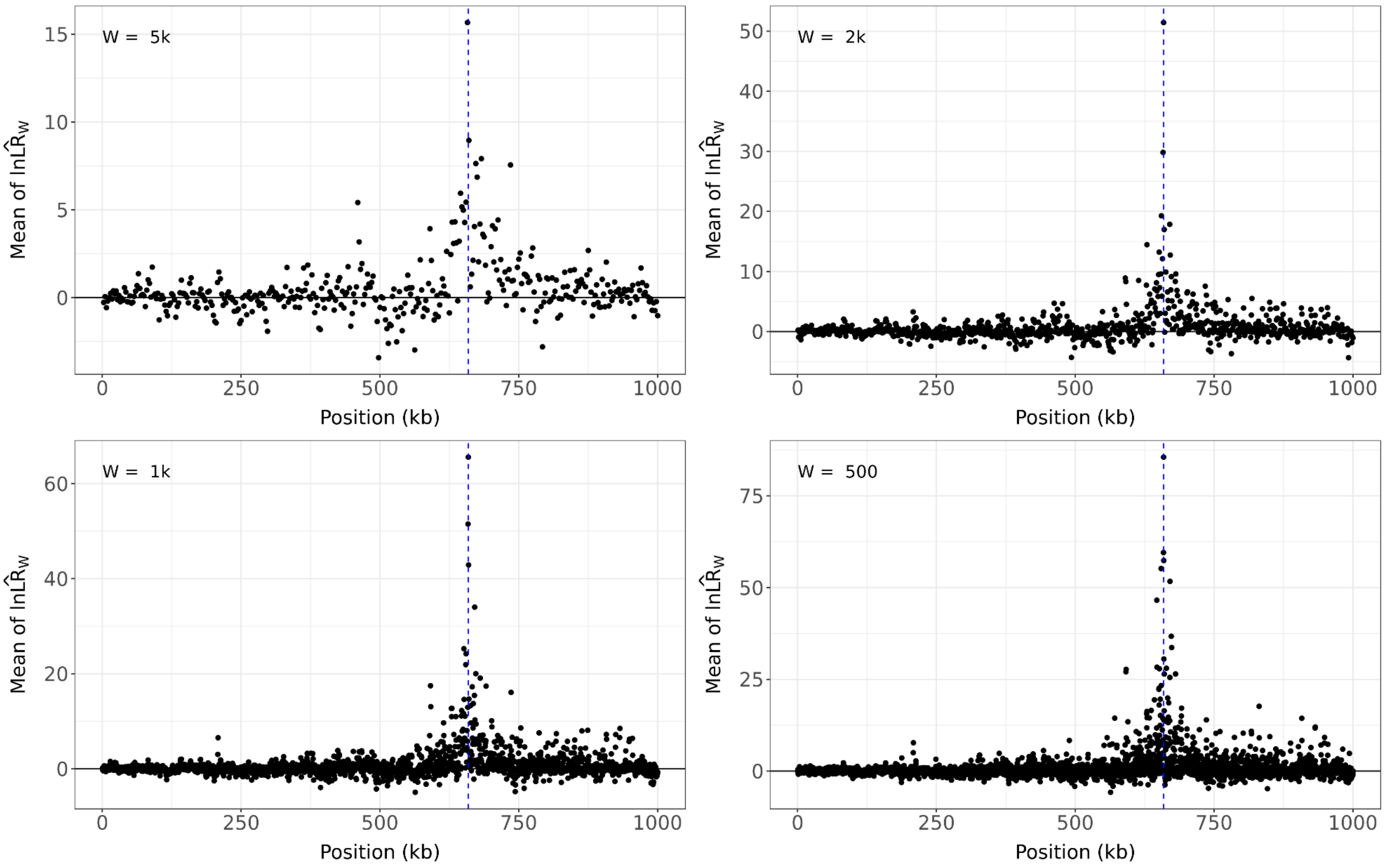

Supplementary Figure 17. Mean of $\ln\widehat{LR}_W$ for n = 1,000, cluster size = 500 bp, and effect size c = 0.6
Each point represents the center position of each of the windows, and the blue vertical line indicates the center of the cluster position.

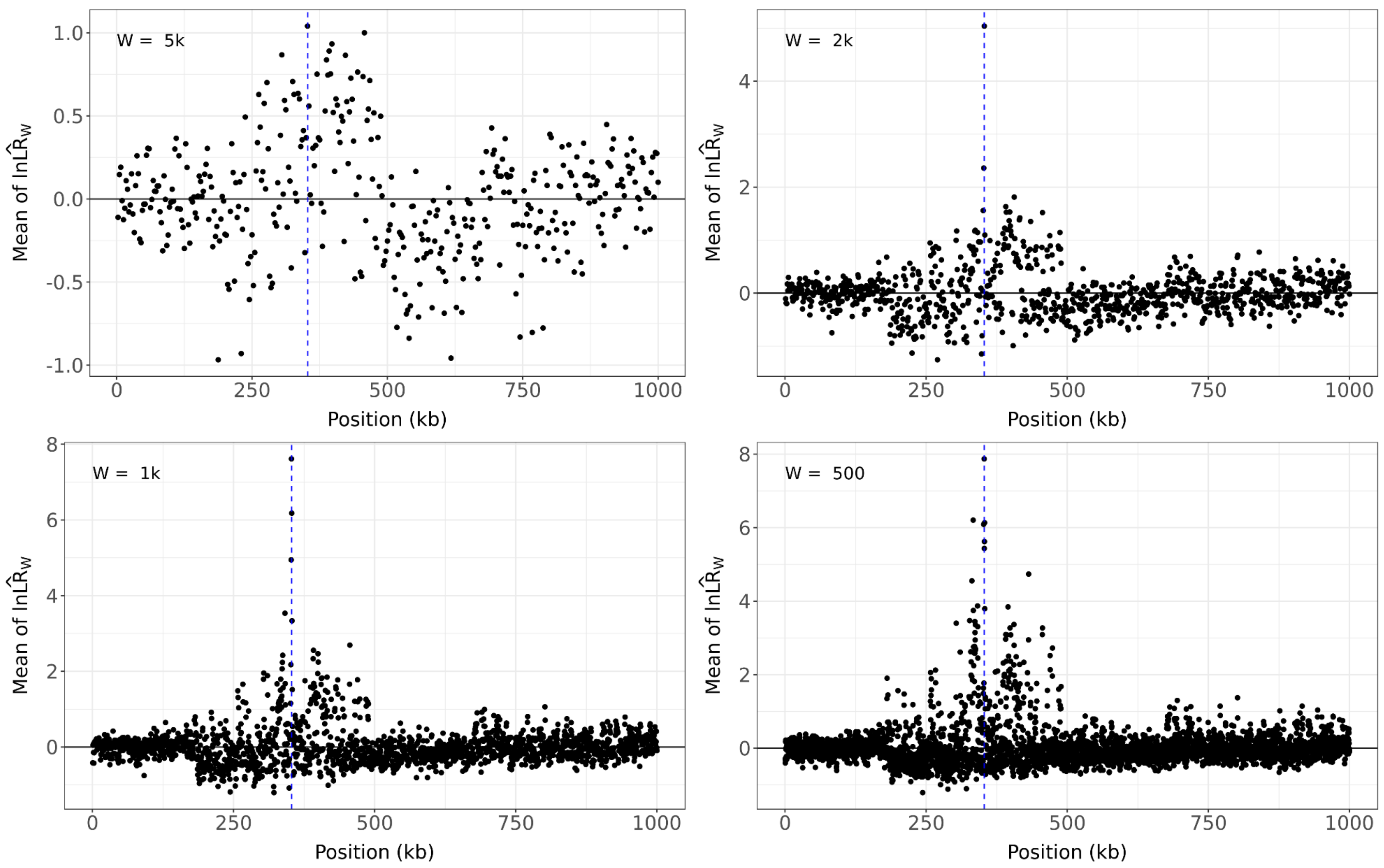

Supplementary Figure 18. Mean of $\ln\widehat{LR}_W$ for n = 1,000, cluster size = 2 kbp (containing 20% disease-related variants), and effect size c = 0.2

Each point represents the center position of each of the windows, and the blue vertical line indicates the center of the cluster position.

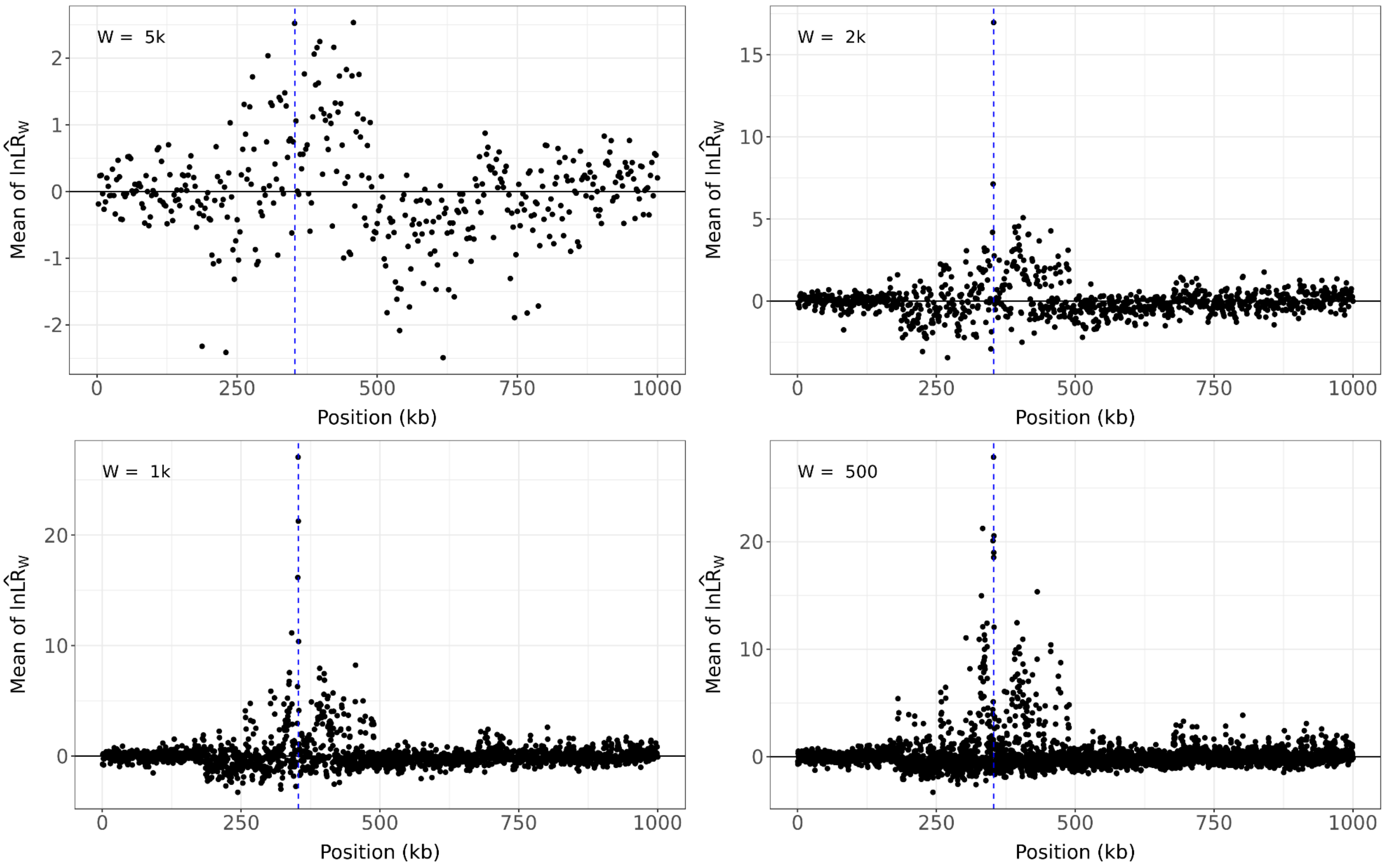

Supplementary Figure 19. Mean of $\ln\widehat{LR}_W$ for n = 1,000, cluster size = 2 kbp (containing 20% disease-related variants), and effect size c = 0.4

Each point represents the center position of each of the windows, and the blue vertical line indicates the center of the cluster position.

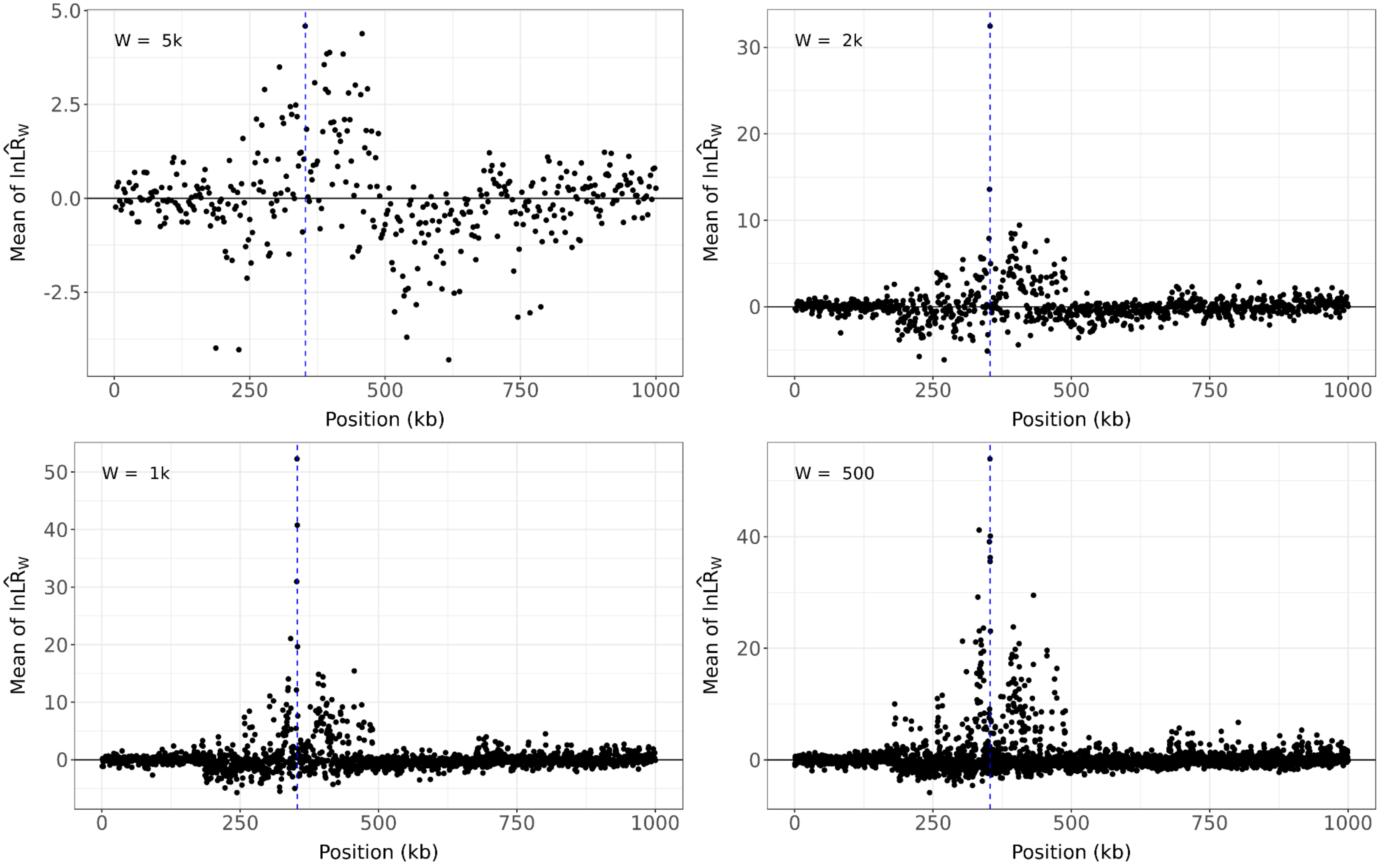

Supplementary Figure 20. Mean of $\ln\widehat{LR}_W$ for n = 1,000, cluster size = 2 kbp (containing 20% disease-related variants), and effect size c = 0.6

Each point represents the center position of each of the windows, and the blue vertical line indicates the center of the cluster position.

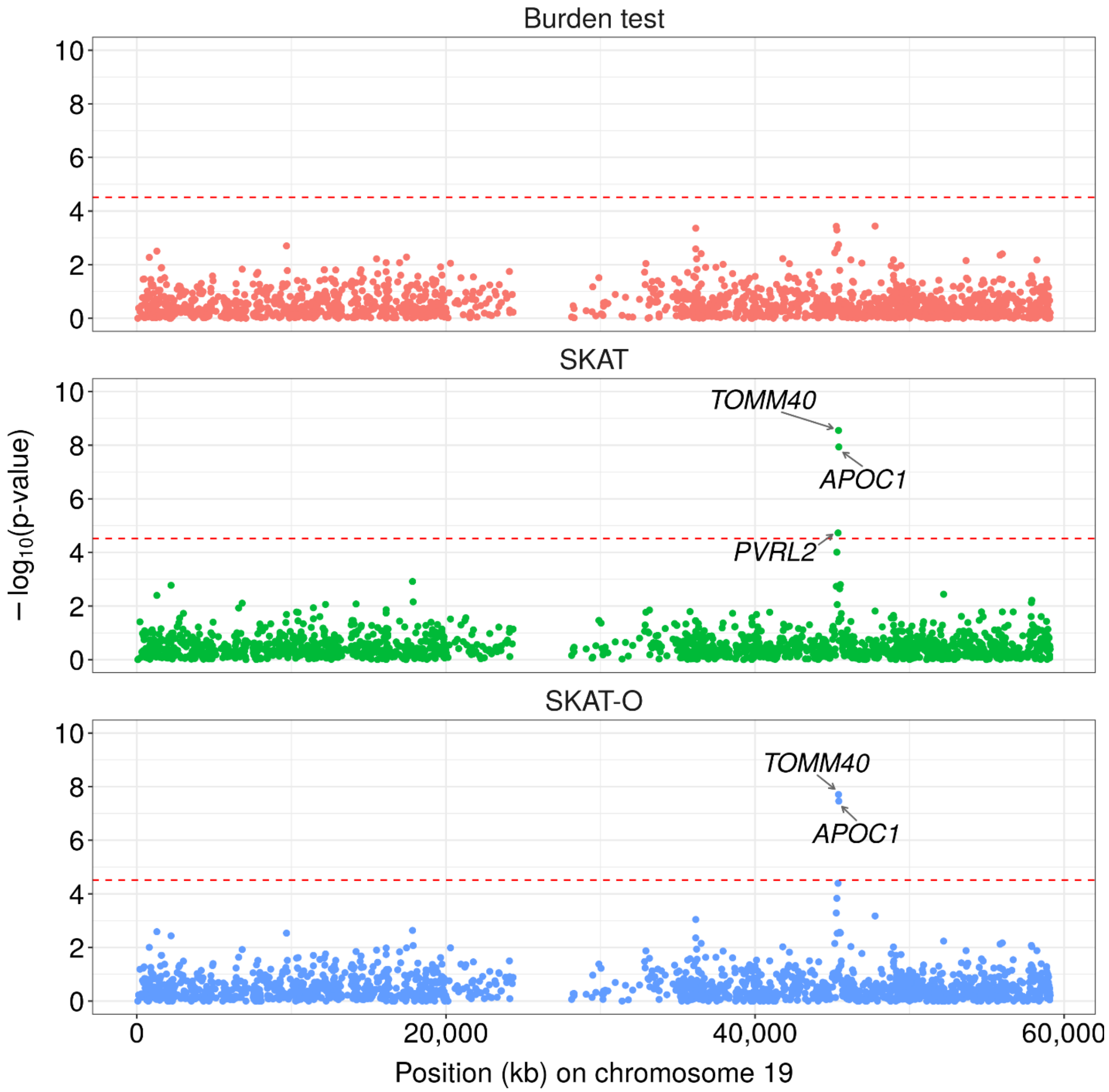

Supplementary Figure 21. Gene-based associations between rare variants located on chromosome 19 and log-transformed CSF amyloid β 1-42 levels in ADNI using the burden test, SKAT, and SKAT-O. The red horizontal line indicates the significance level with Bonferroni correction (α = 0.05/the number of genes on chromosome 19).

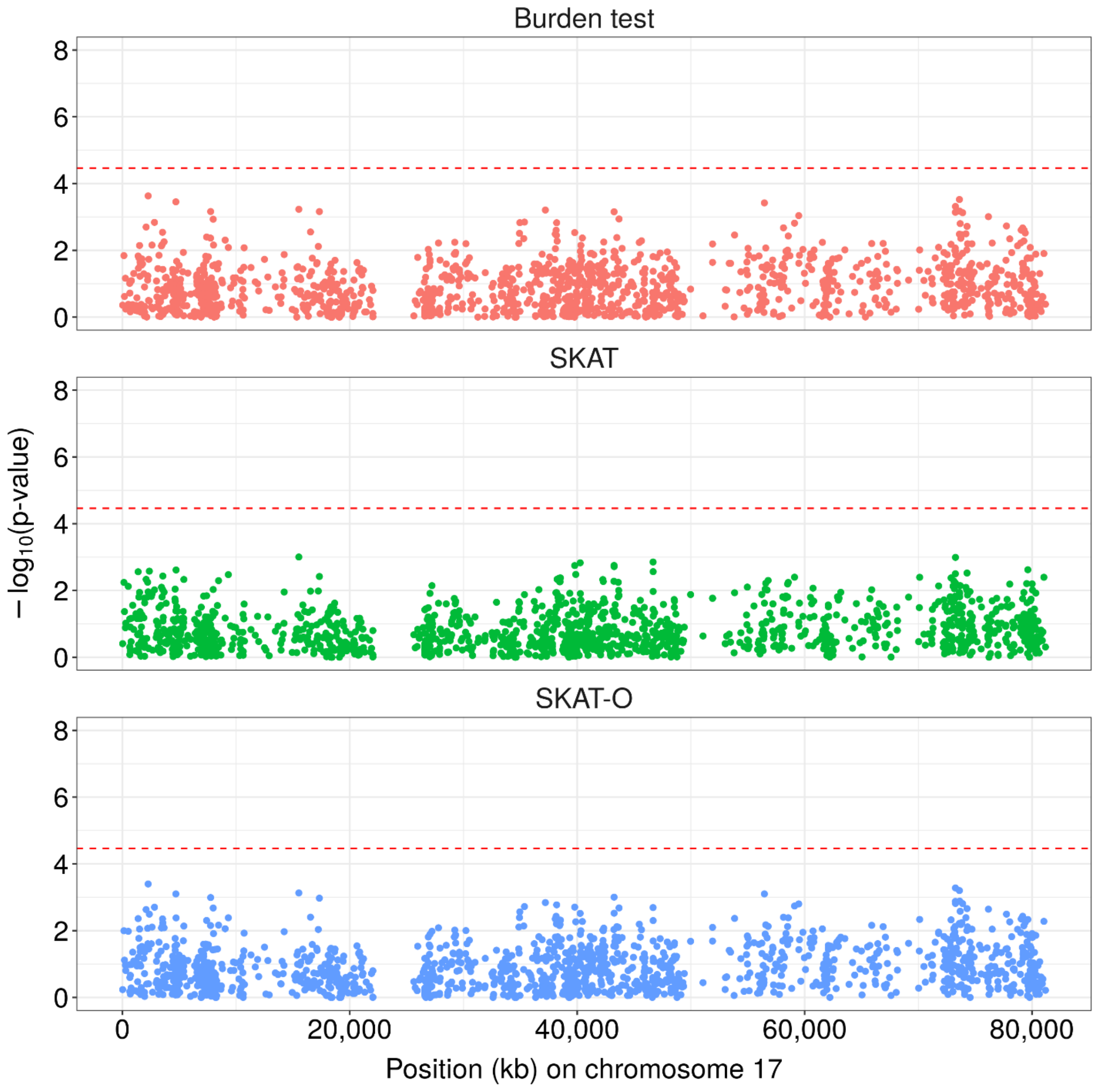

Supplementary Figure 22. Gene-based associations between rare variants located on chromosome 17 and log-transformed CSF phosphorylated tau levels in ADNI using the burden test, SKAT, and SKAT-O. The red horizontal line indicates the significance level with Bonferroni correction (α = 0.05/the number of genes on chromosome 17).

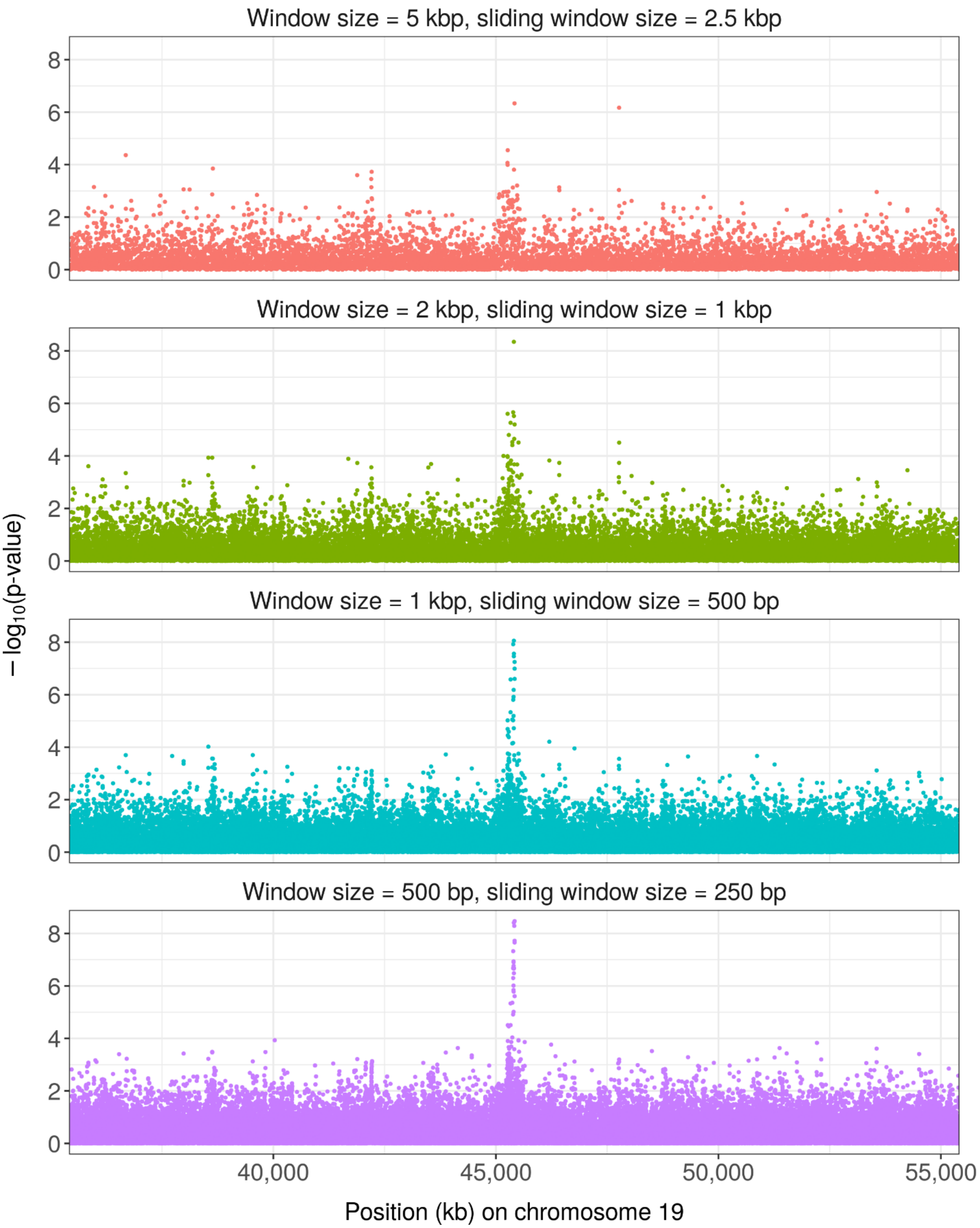

Supplementary Figure 23. QPSS p-values computed by the permutation with generalized Pareto distribution approximation for the associations between rare variants around *APOE* (± 10 Mbp) located on chromosome 19 and log-transformed CSF amyloid β 1-42 in ADNI

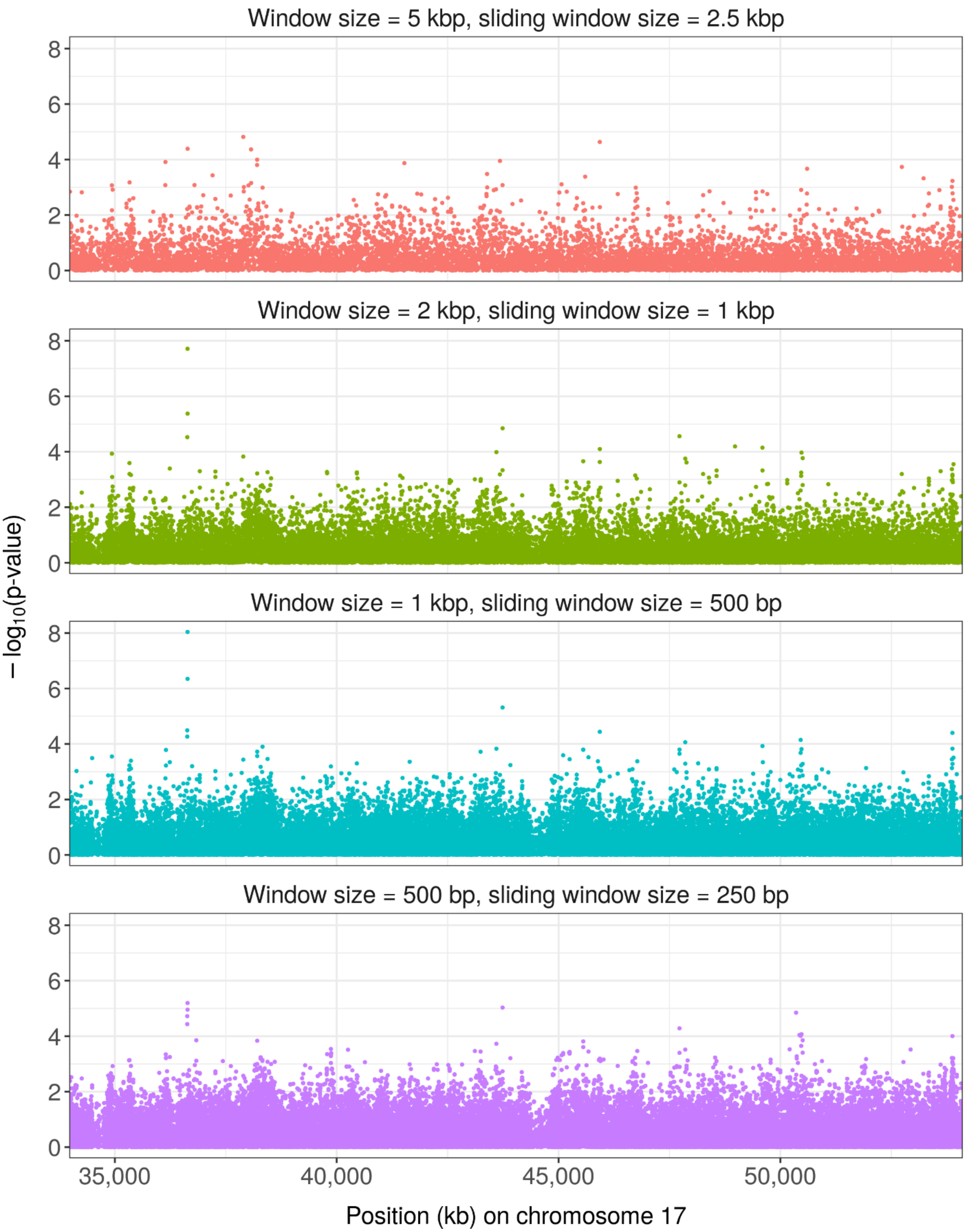

Supplementary Figure 24. QPSS p-values computed by the permutation with generalized Pareto distribution approximation for the associations between rare variants around *MAPT* (± 10 Mbp) located on chromosome 17 and log-transformed CSF phosphorylated tau levels in ADNI

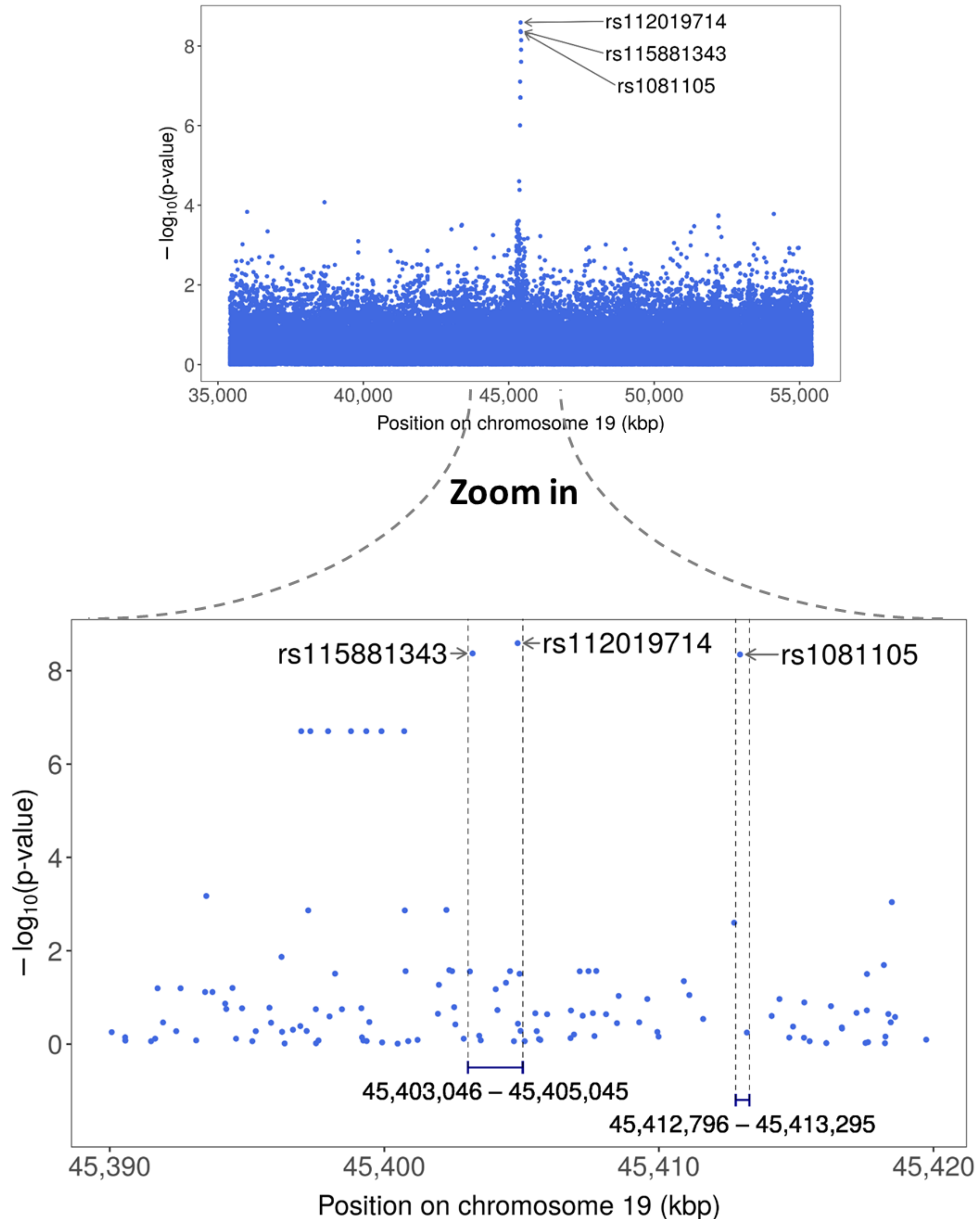

Supplementary Figure 25. Single variant associations between rare variants around *APOE* (± 10 Mbp) located on chromosome 19 and log-transformed CSF amyloid β 1-42 in ADNI
